# Ameliorated Hepatoprotective Aptitude of Novel Lignin Nanoparticles on APAP-Induced Hepatotoxicity in a Murine Model

**DOI:** 10.3390/ph19010071

**Published:** 2025-12-29

**Authors:** Monika Toneva, Nikola Kostadinov, Zhani Yanev, Galina Nikolova, Yanka Karamalakova, Milena Tzanova, Zvezdelina Yaneva

**Affiliations:** 1 Department of Pharmacology, Animal Physiology, Biochemistry and Chemistry, Faculty of Veterinary Medicine, Trakia University, 6000 Stara Zagora, Bulgaria; monika.toneva@trakia-uni.bg; 2 Department of General and Clinical Pathology, Faculty of Veterinary Medicine, Trakia University, 6000 Stara Zagora, Bulgaria; nikola.kostadinov@trakia-uni.bg; 3 Faculty of Industrial Technology, Technical University of Sofia, 1756 Sofia, Bulgaria; zhani@abv.bg; 4 Department of Chemistry and Biochemistry, Faculty of Medicine, Trakia University, 6000 Stara Zagora, Bulgaria; galina.nikolova@trakia-uni.bg (G.N.); yanka.karamalakova@trakia-uni.bg (Y.K.); 5 Department of Biological Sciences, Faculty of Agriculture, Trakia University, 6000 Stara Zagora, Bulgaria; milena.tsanova@trakia-uni.bg

**Keywords:** acetaminophen, hepatotoxicity, morin, lignin nanoparticles, hepatoprotection, nanodelivery

## Abstract

**Background/Objectives**: Acetaminophen (paracetamol or APAP) overdose is a major cause of acute liver injury mediated by oxidative stress, inflammation, and hepatocellular necrosis. The present study investigates the in vivo hepatoprotective potential of morin (M), lignin nanoparticles (LN), and morin-encapsulated lignin nanoparticles (LMN) against APAP-induced hepatotoxicity in mice. The specific goal was to determine whether LMN could strengthen hepatic antioxidant and anti-inflammatory defenses prior to toxic insult, which aligns with a prophylactic model rather than a post-injury clinical rescue approach. This study was guided by the primary hypothesis that LMN pretreatment would markedly reduce APAP-induced hepatic injury. **Methods**: Experimental groups included control, APAP, M, LN, LMN, M+APAP, LN+APAP, and LMN+APAP treatments. Serum hepatic biomarkers, oxidative stress parameters, and inflammatory cytokines were analyzed to assess protective responses. **Results**: APAP exposure markedly elevated aspartate aminotransferase (AST) and alkaline phosphatase (ALP) levels, indicating severe hepatic dysfunction, accompanied by increased lipid peroxidation and pro-inflammatory cytokine production. LMN+APAP treatment significantly restored hepatic enzyme levels to approximately normal values and suppressed malondialdehyde (MDA) formation, while enhancing superoxide dismutase (SOD), catalase (CAT), and glutathione peroxidase (GPx) activities. LMN also downregulated interleukin 6 (IL-6), tumor necrosis factor α (TNF-α), and interleukin 1β (IL-1β), while upregulating interleukin 10 (IL-10), suggesting effective attenuation of inflammatory signaling. Correlation analyses demonstrated positive interactions between MDA, cytokines, and hepatic enzymes, whereas antioxidant enzyme levels were inversely correlated with liver injury markers. Histopathological analysis revealed that treatment with LMN enhanced hepatoprotection, demonstrating predominantly mild, reversible lesions and suggesting a synergistic antioxidant and immunomodulatory effect. **Conclusions**: It could be concluded that LMN provided superior hepatoprotection compared to M or LN. These findings establish LMN as a promising bio-based nanotherapeutic agent for mitigating drug-induced hepatotoxicity through coordinated antioxidant and anti-inflammatory mechanisms.

## 1. Introduction

Acetaminophen (APAP)-induced liver injury persists as a critical clinical concern, stimulating extensive research into advanced therapeutic modalities, including nanoparticle-mediated hepatoprotection [1]. APAP-induced hepatotoxicity is primarily mediated through the formation of the reactive metabolite N-acetyl-p-benzoquinone imine (NAPQI) [2]. Excessive accumulation of NAPQI depletes intracellular glutathione (GSH) reserves, leading to mitochondrial dysfunction, increased production of reactive oxygen species (ROS), and subsequent centrilobular necrosis of hepatocytes. These pathological events are characterized by cellular membrane disruption, enhanced lipid and protein peroxidation, depletion of adenosine triphosphate (ATP), and DNA fragmentation, collectively culminating in oxidative stress and liver failure [3]. Biochemically, APAP-induced hepatotoxicity is associated with marked elevations in serum alanine aminotransferase (ALT), aspartate aminotransferase (AST), and alkaline phosphatase (ALP) levels, together with increased concentrations of malondialdehyde (MDA), a well-established indicator of lipid peroxidation. Concurrently, the activities of major antioxidant defense enzymes, including superoxide dismutase (SOD), catalase (CAT), and glutathione peroxidase (GPx), are significantly reduced, thereby disturbing the cellular redox equilibrium and exacerbating oxidative injury [4,5]. In addition to oxidative processes, the inflammatory response also plays a significant role, characterized by increased levels of TNF-α, IL-1β, and IL-6, while IL-10 exerts anti-inflammatory effects [6]. N-acetyl cysteine (NAC) is the standard antidote used in APAP-induced hepatotoxicity [7]. The development of new effective defense mechanisms is crucial for optimizing the treatment of acetaminophen-induced hepatotoxicity.

The emergence of nanotechnology presents a promising avenue for developing effective solutions to hepatotoxicity and related liver disorders. Advances in nanodelivery systems have enhanced the stability, target specificity, and bioavailability of natural compounds, thereby improving their therapeutic efficacy. Furthermore, progress in genomics and a deeper understanding of genetic determinants underlying liver diseases and the hepatoprotective mechanisms of natural agents have paved the way for personalized therapeutic approaches [8]. In addition, combinatorial strategies involving natural products, biopolymeric carriers, or other bioactive agents have demonstrated improved therapeutic outcomes [9]. Despite encouraging evidence from clinical trials supporting the hepatoprotective potential of natural compounds, several challenges persist in their clinical translation. Consequently, integrating natural compounds with nanotechnology and genomic approaches represents a rational and forward-looking strategy to advance hepatoprotection [8].

In this respect, lignin, a biocompatible and biodegradable natural polymer, has emerged as a promising platform for the design of nanomaterials with therapeutic potential. It has a phenylpropane skeleton with hydrophobic functional groups, consisting of p-hydroxyphenyl (H), guaiacyl (G) and syringyl (S) units, which are derived from the polymerization of hydroxycinnamyl, p-coumaryl, coniferyl, and sinapyl alcohols [10,11]. Due to its rich content of phenolic hydroxyl groups, lignin effectively neutralizes ROS, which play a key role in the pathogenesis of various diseases. In combination with nanotechnology platforms, lignin can be used for the controlled release of various antioxidants, improving their bioavailability and targeted delivery [12]. Recently, lignin nanoparticles (LN) have been considered not only as drug delivery vehicles but also as independent active agents. The combination of lignin-based nanoparticles and antioxidant agents offers a promising approach to reduce drug-induced liver damage. Lignin nanoparticles exhibited excellent liver accumulation. Huo et al. demonstrated in vivo the dose-dependent effect of LN on tumor cells and liver targeting. High doses of LN (300 mg/L) resulted in significant accumulation in tumor cells after intravenous administration. In contrast, doses that are two to five times lower lead to the accumulation of almost all LN in the liver. These results indicate that the liver is the primary site of LN accumulation [13].

Recent studies have shown that nanoparticles can significantly reduce serum liver enzyme markers in APAP overdose. The in vitro studies of Mehta et al. demonstrated that capsaicin-encapsulated lignin nanoparticles considerably reduced the intracellular accumulation of triglyceride as compared to free capsaicin. Their findings suggested that these nanoformulations could serve as a promising therapeutic agent alleviating non-alcoholic fatty liver disease and other chronic inflammatory conditions [14]. The studies of de Souza Porto et al. on hepatocarcinoma and HepaRG cells displayed that orange trunk waste-based LN, encapsulated with curcumin, characterized as nontoxic within 4 h of incubation, and for nanoparticles containing 5% curcumin, toxicity was observed upon application of light in photodynamic therapy experiments, which proved their potential as a therapeutic agent against liver cancer [15].

The therapeutic potential of newly designed eucalyptus and spruce LN against two types of primary liver cancer, hepatocellular carcinoma and cholangio carcinoma in vitro, was reported by Pylypchuk et al. Both types of nanoformulations inhibited the proliferation of hepatocellular carcinoma (HCC) cells in a dose-dependent manner and did not affect cholangio carcinoma (CCA) cell line growth. The authors proposed that the elevated number of nanoparticle-surface carbohydrates was responsible for the significant mediation of the interaction between LN and eukaryotic cells [16].

The review of contemporary scientific literature identifies lignin-based nanoformulations as promising platforms for a variety of targeted therapeutic applications. Nevertheless, there remains a notable scarcity of in vivo studies exploring the hepatoprotective potential of nanoparticles derived from the heterobiopolymer. Given the inherent propensity of lignin to accumulate in hepatic tissue, this characteristic may be strategically exploited in animal models of drug-induced hepatotoxicity. Moreover, the phenolic structure and intrinsic antioxidant properties of lignin nanoparticles are expected to mitigate oxidative stress and support hepatocellular defense mechanisms [17]. The proven capacity of lignin nanoparticles to encapsulate bioactive compounds further enables the design of multifunctional therapeutic systems with enhanced effectiveness [18]. The latter outcomes provoked the aim of the present study to investigate the in vivo hepatoprotective potential of lignin and morin-encapsulated lignin nanoparticles in mitigating APAP-induced hepatotoxicity in mice. Our specific goal was to determine whether LMN could strengthen hepatic antioxidant and anti-inflammatory defenses prior to toxic insult, which aligns with a prophylactic model rather than a post-injury clinical rescue approach. The primary hypothesis of this study was that pretreatment with morin-encapsulated lignin nanoparticles (LMN) would confer superior hepatoprotection compared to free morin (M) or lignin nanoparticles (LN) alone. We specifically hypothesized that LMN pretreatment would significantly attenuate APAP-induced hepatic injury, reflected by reductions in serum alanine aminotransferase (ALT) and aspartate aminotransferase (AST) levels. Accordingly, serum ALT and AST were designated as the predefined primary endpoints, while oxidative stress markers, antioxidant enzyme activity, inflammatory cytokine profiles, and histopathological evaluation served as secondary endpoints to further elucidate the mechanistic basis of LMN-mediated protection.

## 2. Results and Discussion

### 2.1. ξ-Potential, TEM, FTIR, and XRD Characteristics of LN and LMN

The experimentally determined ζ-potential values of LN and LMN are summarized in Table 1. A commonly accepted threshold distinguishing stable from unstable colloidal suspensions is ±30 mV; particles with ζ-potential values greater than +30 mV or less than −30 mV are typically considered stable and strongly cationic or anionic, respectively [19]. The ζ-potential also influences the ability of nanoparticles to interact with and permeate cellular membranes. In general, cationic particles exhibit higher toxicity due to their stronger interactions with negatively charged cell membranes [19]. Based on these criteria, both types of the studies of lignin-based nanoformulations could be classified as anionic, with the unloaded formulation displaying a more pronounced anionic character. Moreover, both nanoparticle types fall within the range associated with good colloidal stability.

**Figure 1:**
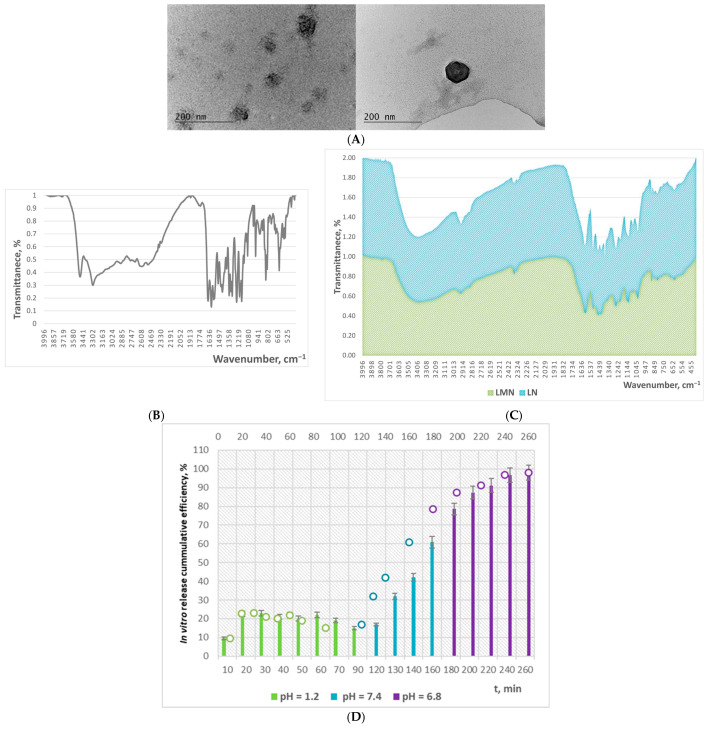
(**A**) TEM images of LN; (**B**) FTIR spectrum of morin; (**C**) FTIR spectrum of LN and LMN; (**D**) in vitro release kinetics curve of morin from LMN in simulated gastrointestinal medium.

The TEM images of LN, presented in Figure 1A, display that the nanoparticles possess a homogeneous, spherical, and well-defined morphology. The particle size ranges, summarized in Table 1, for both blank and morin-encapsulated lignin nanoparticles indicate an increase in particle diameter upon encapsulation of the bioactive compound. Smaller nanoparticles (<100 nm) generally exhibit enhanced cellular uptake and more favorable biodistribution profiles, which can facilitate improved delivery to hepatic tissues.

The XRD diffractogram of LN, presented in [18], characterized with a broad peak, indicating that they characterize with poor crystallinity.

The absorbance peak in the FTIR spectrum of LN and LMN (Figure 1C) at 3370 cm^−1^ corresponds to the total O–H groups, while the peak at 2930 cm^−1^ is associated with C–H stretching in –CH_3_, –CH_2_, and –OCH_3_ groups. The band at 1600 cm^−1^ can be attributed to C=O stretching vibrations. The region 1600–1500 cm^−1^ is characteristic of aromatic ring vibrations and C–H, O–H, and C=O bond contributions. The peaks at 1460, 1420, 1320, and 1260 cm^−1^ correspond to O–H in-plane bending; aromatic ring vibrations combined with C–H in-plane bending; bending of C–H and C–O bonds; and C–O–C bond vibrations, respectively. Both spectra exhibited a band at 1134 cm^−1^, which is indicative of condensed phenolic groups [18]. The characteristic double peak at 3520 and 3300 cm^−1^ in the FTIR spectrum of morin corresponds to O–H stretching vibrations associated with flavonoid dimers (Figure 1B). Bands within the 1230–1210 cm^−1^ region are associated with syringyl unit bending, whereas those at 1300, 1190–1000, and 890 cm^−1^ correspond to stretching vibrations of guaiacyl structures [20]. Generally, although some peaks exhibited slight shifts in wavelength, no new characteristic peaks appeared in the spectrum of the flavonoid-loaded nanoparticles. The latter is associated with successful encapsulation of morin within the lignin nanoparticle network without undergoing unintended chemical reactions with the heteropolymer [20].

### 2.2. In Vitro Release Potential

Morin loading was highly efficient, achieving 98.1% encapsulation capacity. In vitro release studies demonstrated pH-dependent behavior, with LMN showing the highest morin release (~24%) at pH 6.8, approximately double that observed at pH 7.4 and triple that at pH 1.2 (Figure 1D).

### 2.3. In Vivo Study

The purpose of administering APAP at 200 mg/kg daily for three consecutive days in the present experimental model was to establish chronical hepatotoxicity. Using a 3-day repeated APAP dosing at 200 mg/kg/day is intended to create a sub-acute/early chronic injury model, especially when considering that chronic-dosing studies often use repeated administrations over days to weeks to model progressive liver stress or cumulative toxicity; and lower daily doses, compared to single large overdose, more closely mimic repeated overdosing or therapeutic misuse, which is clinically relevant. In this respect, Ding et al. report a dose of 400 mg/kg APAP i.p. twice a day, which means that the final dose is 800 mg/kg that corresponds to the dose administered in the present experiment [21]. The study of Al-Doaiss et al. demonstrates that prolonged repeated weekly APAP dosing is used to model chronic liver stress rather than a single overdose, supporting the conceptual validity of a repeated-dose model for “chronic hepatotoxicity.” [22]. Aging, nutritional status, and repeated APAP dosing, as reported by Kane et al., can modulate susceptibility, indicating that “sub-acute/chronic” hepatotoxic models do not always rely on extremely high single doses [23]. The detailed review of Mao et al. collates various protocols, implying a spectrum of APAP-induced liver injury models from acute overdose to sub-chronic dosing, underlining that a 3-day, 200 mg/kg/day regimen fits within the broader landscape of in vivo APAP studies [24].

In this study, all formulations (M, LN, and LMN) were intentionally administered as pretreatments to evaluate their preventive hepatoprotective potential, rather than their therapeutic efficacy after injury. Our goal was to determine whether LMN could strengthen hepatic antioxidant and anti-inflammatory defenses prior to toxic insult, which aligns with a prophylactic model rather than a post-injury clinical rescue approach. As such, we did not include a positive control group treated with N-acetylcysteine (NAC), which serves as a standard therapeutic intervention used after APAP overdose in clinical settings. Because our focus was on prophylactic protection, not therapeutic rescue, NAC was not appropriate for the specific aims of the present study.

This preventive study design is consistent with established methodologies used in previous hepatoprotection studies, including those by Papáčková et al. (2018) and Chowdhury et al. (2019), which similarly evaluated natural compounds or nanoformulations as pretreatment-based protective agents against APAP toxicity [25,26].

The 50 mg/kg dose of morin, LN, and LMN was selected based on prior studies using flavonoid-loaded polymeric nanoparticles in mice, where doses between 25 and 100 mg/kg are commonly employed for antioxidant or hepatoprotective effects. At this range, lignin-based nanoparticles have shown good tolerability with no systemic toxicity in vivo. While direct clinical translation cannot be inferred, the administered dose falls within the typical exploratory range for murine nanotherapeutic studies. Higher doses were not evaluated here, but published work indicates that lignin nanoparticles remain well tolerated up to several hundred mg/kg, suggesting a reasonable safety margin. Huo et al. administered up to 300 mg/kg of lignin nanoparticles intravenously in mice and reported good tolerability. This supports that lignin-based NPs can be tolerated at doses well above 50 mg/kg [27]. Flavonoid-loaded zein NPs, dosed at 25 mg/kg every 48 h in mice, improved bioavailability and produced biological effect without reported toxicity [28].

#### 2.3.1. Attenuation of APAP-Induced Acute Liver Injury in Mice

AST and ALT are two hepatic-specific enzymes that reflect the degree of acute liver damage, and they are frequently elevated after excessive alcohol intake [29]. When liver cells are damaged, cell membrane permeability increases, and AST and ALT are released into the blood, increasing serum transaminase content, which is the essential enzyme in metabolic processes [30]. Alkaline phosphatase (ALP) is a membrane-bound enzyme but not a liver-specific enzyme, and elevated levels of ALP may reflect impaired biliary function or various liver diseases, including cholestasis, which is a condition where bile flow is blocked [31].

**Figure 2:**
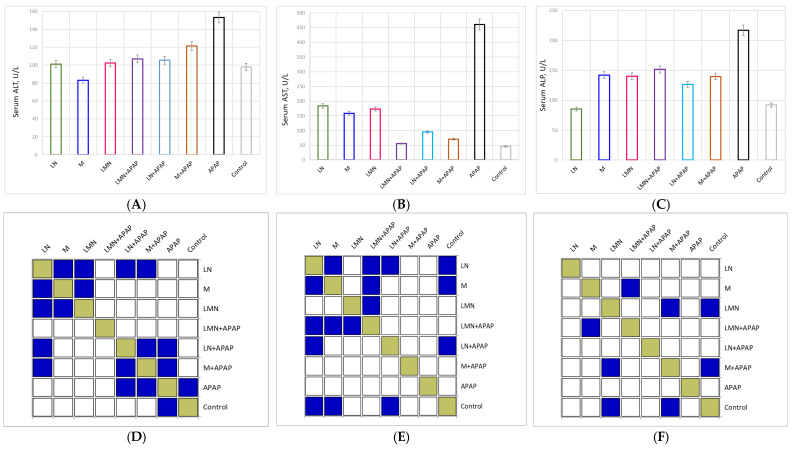
Effects of morin, lignin nanoparticles (LN), and morin-loaded lignin nanoparticles (LMN) on serum ALT (**A**), AST (**B**), and ALP (**C**) levels in APAP-induced hepatotoxic mice (mean ± SD, *n* = 6); Tukey post hoc *p*-value maps (*p* < 0.05) of (**D**) ALT; (**E**) AST; and (**F**) ALP highlighting key group differences. AST differences mainly involve LN and morin treatments; ALT shows significant contrasts among M, LMN, and M+APAP; and ALP reveals broader shifts, with LN, M, LMN, and their APAP combinations differing significantly from several groups. Statistically significant difference—*p* < 0.05 marked in blue.

The influence of APAP-induced toxicity and the protective potential of pure morin and lignin nanoparticles and morin-encapsulated nanoparticles alone and followed by APAP treatment on the hepatic enzymes’ ALT, AST, and ALP levels in the blood serum of the experimental animals are shown in Figure 2.

In blood serum, the activities of AST and ALT of mice in the control group were 45.7 U/L and 98.0 U/L, respectively, while those in the APAP group were 460.7 U/L and 153.3 U/L. Morin alone was characterized by the highest positive effect on the determined ALT values (83.2 U/L) among all the other experimental groups. Its value was even lower than that of the control group. A decrease in ALT concentration was also established in the LNP-treated group (101.1 U/L), but to a lesser extent. The experimental results outlined that the administration of APAP to the groups treated with LMN and LN in advance lead to an insignificant increase in the concentration of the liver enzyme. Statistically significant deviation was encountered only between the M-treated and M+APAP-treated groups.

The control group exhibited normal baseline enzyme activities (AST: 45.7 U/L; ALT: 98 U/L; and ALP: 92 U/L), reflecting healthy liver function. In contrast, the APAP-treated group showed a dramatic increase in all three enzymes (AST: 460.7 U/L; ALT: 153.3 U/L; and ALP: 216.8 U/L). These elevations represent approximately 10-fold (AST), 1.6-fold (ALT), and 2.4-fold (ALP) increases relative to control, consistent with acute hepatocellular injury and cholestatic dysfunction. This confirms that the APAP model successfully induced significant hepatic damage, primarily due to oxidative stress and glutathione depletion leading to necrosis.

The groups receiving morin, LN, or LMN alone showed enzyme levels similar to the control, confirming the non-toxic nature of these treatments. Morin alone caused no significant enzyme elevation, indicating hepatic safety. LN alone (AST: 184 U/L) remained within physiological limits, confirming biocompatibility of lignin nanoparticles. LMN alone also showed no hepatotoxic effect. Thus, none of the formulations alone compromised liver integrity, suggesting suitability for therapeutic application.

Morin co-administration with APAP reduced enzyme levels: AST decreased by 85%, ALT by 21%, and ALP by 36% compared to APAP alone. The registered reduction indicates that the bioflavonoid provides moderate hepatoprotection, likely via its known antioxidant and free-radical scavenging activity, restoring membrane integrity and preventing enzyme leakage. In the LN+APAP group, enzyme levels were lower than in the APAP group, especially the AST values. The reduction in the enzyme concentrations, by 79% for AST, 31% by ALT, and 42% by ALP, imply that lignin nanoparticles alone exhibit inherent hepatoprotective effects, possibly due to phenolic structures in lignin that neutralize reactive oxygen species (ROS). The combination LMN+APAP yielded profound normalization of enzyme activities with AST levels (55.5 U/L) nearly matching the control (only ~21% higher) and with 88% lower than the APAP group. Besides both ALT (107 U/L) and ALP (151.5 U/L) were reduced by approximately 30% as compared to APAP, indicating significant hepatocellular recovery. This enhanced effect of free morin and LN suggests additive effect of the antioxidant properties of both components. The sustained release of morin from degradation in the LMN formulation, established previously by our scientific team in simulated gastrointestinal medium, likely allow for prolonged antioxidant action, effectively mitigating APAP-induced oxidative injury [32].

The enhanced protection of LMN can be attributed to the following: (i) improved solubility and stability of morin when nanoencapsulated; (ii) controlled and sustained release, ensuring prolonged therapeutic concentration in hepatocytes; (iii) enhancement of the antioxidant activity due to lignin phenolic groups and morin flavonoid structure; and/or (iv) better cellular uptake of LMN due to their nanoscale size, enhancing intracellular delivery and ROS scavenging.

Similar results for the protective potential of lignin derivatives were reported in the study of Ren et al. They examined the protective effect of flaxseed lignans on liver damage caused by an overdose of paracetamol. The findings demonstrated that administering 800 mg/kg/d flaxseed lignan prior to paracetamol significantly decreased the serum aspartate AST, ALT, and total bilirubin levels, while it increased liver superoxide dismutase (SOD) and glutathione (GSH) levels in mice [33].

The hepatoprotective effect of syringic acid (SA), one of lignin structural units, against sodium valproate (SV)-induced liver injury in rats was studied. SV administration for 14 days caused significant elevation of liver transaminases and ALP in serum. The experimental results proved the potential applicability and effectiveness of SA as a promising herbal drug that can inhibit sodium valproate-induced hepatotoxicity when administered together due its hepatoprotective, potential anti-inflammatory activity [34]. Lignin also has demonstrated remarkable antioxidant and liver-parenchyma regenerative properties making it a suitable polymer for the development of delivery cargo in treating non-alcoholic fatty liver disease [35]. Moreover, lignin nanoparticle-treated HepG2 cells demonstrated a substantial protein reduction indicating that LNP alone might exert a mild protective effect against osteoarthritis-induced stress by interacting with cellular components to mitigate damage. In vitro studies demonstrated that capsaicin-loaded lignin nanoparticles substantially reduced intracellular accumulation of triglyceride compared with free capsaicin, and thus they can be a promising therapeutic approach that can contribute to the treatment of liver-related diseases and other chronic inflammatory conditions [14].

Morin has demonstrated significant hepatoprotective effects through its ability to attenuate oxidative stress, suppress pro-inflammatory cytokines, and regulate apoptotic pathways in hepatocytes. Multiple cellular targets and signaling pathways have been shown to be modulated by morin, indicating its multifaceted protective profile [36]. Morine administration to Sprague-Dawley rats with induced hepatic ischemia/reperfusion in two doses, 50 and 100 mg/kg, proved its shielding activity by improving the histopathological deterioration and lessening the serum ALT and AST levels in a dose-dependent manner [37]. The study of Bhakuni et al. proved the beneficial role of morin in the treatment of alcohol-induced hepatotoxicity in rats by inhibition of oxidative stress and inflammatory mediators [31]. Moreover, the results of Sheoran et al. showed that morin significantly attenuated lead-induced hepatotoxicity as indicated by reduced lipid peroxidation and protein oxidation in biochemical assays [38]. Shali et al. explored the modulatory effect of morin on oxidative stress and carbohydrate metabolism in the liver of streptozotocin (STZ)-induced diabetic rats. The results outlined that morin effectively modulated the alternations in the concentration of lipid peroxidation products, activities of antioxidant enzymes and carbohydrate metabolizing enzymes in the liver of the diabetic rats. The overall potential was comparable with this for diabetic rats administered with metformin [39]. The results of Folorunso et al. displayed that normal rats, Type 2 diabetic rats, and diabetic rats exposed to diesel exhaust particles exhibited a substantial decrease in oxidative stress indicators, serum lipid profile, and levels of AST and ALP, as well as an increase in liver natural antioxidants following oral administration of morin [40].

The hepatoprotective activity of the novel morin-vitamin E-β-cyclodextrin inclusion complex loaded chitosan nanoparticles (M-Vit.E-CD-CS NPs) designed by Mondal et al. was examined against arsenic-induced toxicity in a murine model. The values of ALT and AST outlined potency was nearly 10 times higher of the M-Vit.E-CD-CS NPs than morin and 2.5 times higher than vitamin E. Consequently, the effect of M-Vit.E-CD-CS NPs treatment showed better effects than free morin or vitamin E in all cases [41].

#### 2.3.2. Attenuation of APAP-Induced Oxidative Stress in Mice

**Figure 3:**
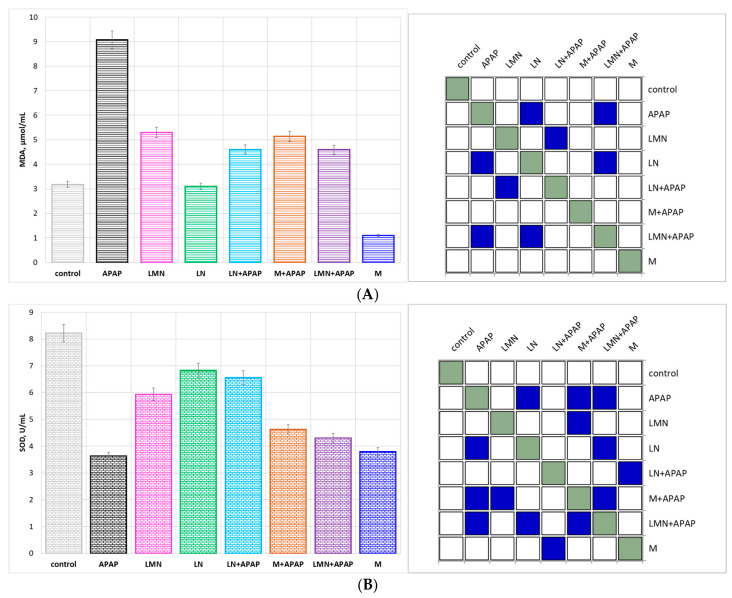

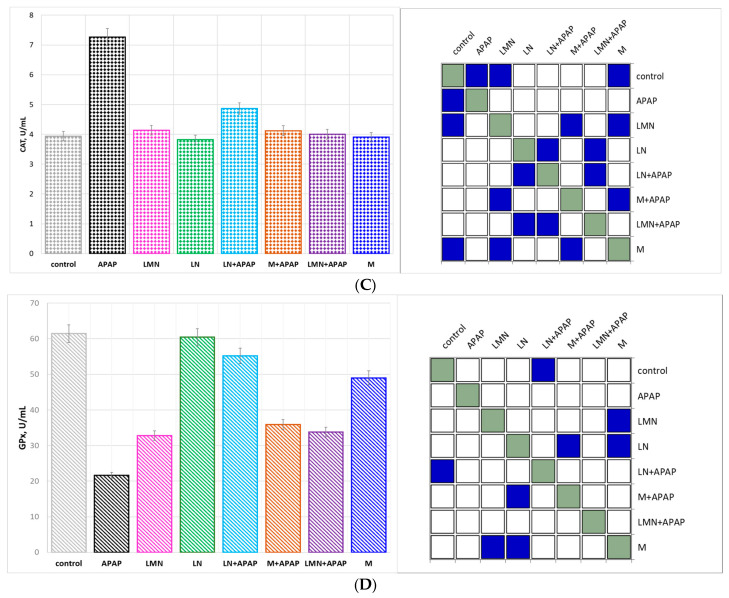
Morin, LN, and LMN modulation of APAP-induced oxidative stress in hepatic samples. (**A**) MDA activity and Tukey post hoc *p*-value correlation map of MDA; (**B**) SOD activity Tukey post hoc *p*-value correlation map of SOD; (**C**) CAT activity and Tukey post hoc *p*-value correlation map of CAT; (**D**) GPx levels and Tukey post hoc *p*-value correlation map of GPx. Values represent the mean ± SD (*n* = 6). Statistically significant difference—*p* < 0.05 marked in blue.

Based on the association of oxidative stress markers with hepatic injury following APAP overdosing, the MDA, SOD, CAT (Figure 3A), and GPx (Figure 3B) levels in liver homogenates of mice were determined. Generally, abnormal SOD and CAT levels in the cell causes the accumulation of intracellular ROS, which leads to lipid peroxidation and the production of MDA, which can cause cell membrane damage [42].

The control group displayed normal baseline values for oxidative stress markers: low MDA (3.18 µmol/mL) values and high antioxidant enzyme activities: SOD 8.22 U/mL, CAT 3.94 U/mL, and GPx 61.43 U/mL, reflecting intact hepatic redox homeostasis. Upon APAP administration, there was a profound alteration in this balance, resulting in 3-fold MDA increase up to 9.07 µmol/mL, accompanied by a 56% drop in SOD and a 65% reduction in GPx activities. These changes confirm the occurrence of severe oxidative stress, resulting probably from the accumulation of N-acetyl-p-benzoquinone imine (NAPQI)—a toxic metabolite product of acetaminophen. In high doses of APAP administration NAPQI can accumulate in the liver and cause hepatotoxicity by depleting glutathione and forming protein adducts within mitochondria, which impair the mitochondrial respiratory chain, leading to increased superoxide generation and reduced ATP production [43]. The registered approximately 2-fold elevated CAT activity (7.26 U/mL) probably represents a compensatory response to excessive H_2_O_2_ generation.

The formulations administered without APAP induced neither oxidative stress nor significant enzyme alterations, which serves as a confirmation of their biocompatibility. Morin alone significantly reduced the MDA activity of the control group by 65%, confirming its intrinsic antioxidant activity and potential to inhibit lipid peroxidation. The SOD value of the flavonoid slightly below the control could be attributed to bioactivity based on non-enzymatic radical scavenging rather than to enzymatic stimulation. The intrinsic antioxidant potential of lignin nanoparticles, associated with the multiple phenolic hydroxyl groups in the structure of lignin capable of donating H^+^ to neutralize free radicals, was proved by the registered MDA, SOD, and GPx levels close to these of the control group. The observations regarding the potential of LMN alone indicate that the morin-loaded nanoparticle formulation is non-toxic and can maintain hepatic oxidative balance. Besides, it could be assumed that, under non-stressed conditions, morin release from the lignin matrix is gradual and lower than its maximal capacity.

The co-treatment with morin/LN/LMN followed by APAP overdose administration provoked significant biochemical improvements. All co-administered groups exhibited substantial MDA decrease, which accounted for 49% for the LN+APAP and LMN+APAP groups and approximately 43% for the M+APAP group compared to the APAP-only group.

SOD activity was recovered by 27% in the M+APAP and by 18% in the LMN+APAP groups. The strongest SOD restoration extend was registered in the LN+APAP group (81%), suggesting that lignin nanoparticles most effectively enhance superoxide detoxification. This may result from lignin phenolic structures acting as SOD mimetics or stimulating endogenous antioxidant enzyme expression. APAP treatment led to extensive elevation of CAT to a value of 7.26 U/mL, associated with oxidative overloading. CAT activity was reduced to approximately normal values in the M+APAP, LN+APAP, and LMN+APAP groups, which suggests the balancing of ROS metabolism and establishment of a stable redox environment under reduced oxidative stress.

Within the vital redox network of mammalian cells, GPx represent a prominent enzyme family with diverse functions that influence nearly all cellular processes. GPx is a crucial parameter for detoxifying H_2_O_2_ and maintaining glutathione balance (GSH) [44]. The LN+APAP group displayed the highest recovery, which is indicative of the potential of lignin nanoparticles to enhance the glutathione-dependent antioxidant system, either by GPx synthesis regulation or through the indirect preservation of glutathion reserves. The co-treated group LMN+APAP characterized with moderate GPx recovery of 56%. The latter could possibly be explained by the controlled release kinetics of morin, which can be described as sufficient to limit peroxidation but not yet optimal to ensure complete recovery of GPx.

Among all tested nanoparticle formulations, the co-administration of lignin nanoparticles and APAP provoked the most consistent enzyme recovery and MDA suppression, followed closely by the group LMN+APAP. The group treated with the flavonoid and APAP (M+APAP) exhibited moderate protection activity. However, the LMN+APAP group displayed the lowest lipid peroxidation and the most balanced CAT normalization, which is evincible for oxidative processes effective control, although fewer fluctuations in enzyme activities were registered. The latter observations represent a sustained and more physiologically regulated antioxidant response due to controlled morin release and additive effect with the lignin matrix.

Similar but scarce results were obtained by other scientific teams regarding the antioxidant potential of morin and lignin-based formulations. Sheoran et al. stated that morin significantly enhanced the activities of SOD and CAT, as well as elevated glutathione levels in rats exposed to lead. Molecular docking results confirmed a strong binding affinity between morin and both SOD and CAT, reinforcing the role of the bioflavonoid in strengthening antioxidant defense mechanisms. Moreover, morin effectively prevented lead-induced liver damage, such as hydropic degeneration, portal vein congestion, and collagen fiber accumulation, as evidenced by histological and ultrastructural analyses. Overall, the subchronic study demonstrated the strong therapeutic and antioxidative potential of morin in protecting hepatic tissue from lead toxicity [38].

Deng et al. reported that lignin nanoparticles obtained by high-pressure homogenization technology exhibited higher in vivo and in vitro antioxidant activity as compared to original craft lignin, which could endow it with enhanced protective effects for the vascular and neural development of bisphenol AF-induced zebrafish. LN outperformed craft lignin in terms of the higher degree of restoration of SOD and CAT activities to normal levels [45].

The therapeutic effect of implantable and degradable antioxidant poly(ε-caprolactone)-lignin nanofiber membrane for effective osteoarthritis treatment was studied by Liang et al. The obtained results indicated that the lignin nanofibers were able to assist chondrocytes to resist oxidative stress, which was sustained by the 70–80% increased SOD and CAT activities and the 40% decrease in the MDA levels [46].

The review of recent scientific literature did not find studies investigating and assessing in vivo the activity of lignin nanoparticles and morin-loaded lignin nanoparticles to ameliorate the oxidative stress provoked by drugs-induced hepatotoxicity—a fact that emphasizes indisputably the novelty and scientific significance of the present study.

#### 2.3.3. Suppression of APAP-Induced, Hepatic Inflammatory Response in Mice

**Figure 4:**
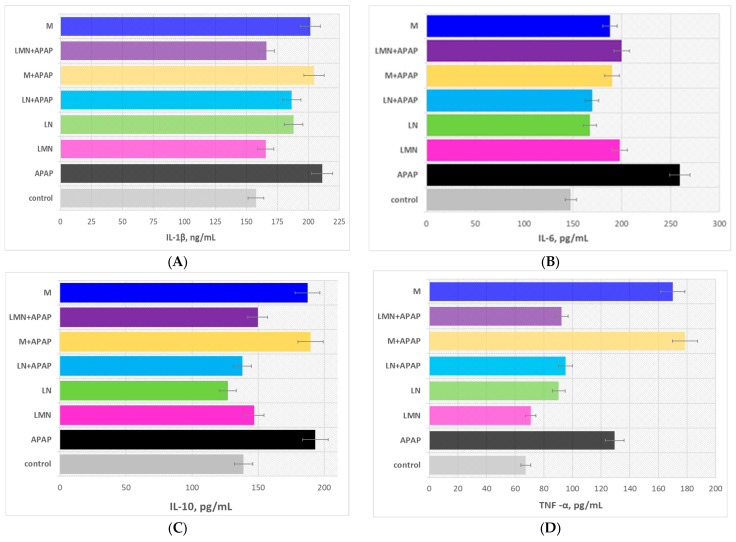
Effect of morin and LN and LMN alone and in combination with APAP on the levels of APAP-induced hepatic inflammatory cytokines in mice: (**A**) the pro-inflammatory cytokine IL-1β; (**B**) the pro-inflammatory cytokine IL-6; (**C**) the anti-inflammatory cytokine IL-10; and (**D**) the pro-inflammatory cytokine TNF-α. Values represent the mean ± SD (*n* = 6).

Inflammatory responses play a pivotal role in inducing liver injury. In this study, we assessed the release of three critical pro-inflammatory cytokines, TNF-α, IL-1β, and IL-6, and the anti-inflammatory cytokine IL-10 in the serum of mice using ELISA kits. The experimental results are presented in Figure 4, and the statistical significance of the data was evaluated by the data in Table 2.

The results clearly demonstrate that APAP administration significantly disrupted the hepatic cytokine balance, reflected by the substantial elevation of the pro-inflammatory mediators IL-6, TNF-α, and IL-1β and by the compensatory increase in the anti-inflammatory cytokine IL-10. The treatment with LN or LMN alone did not provoke inflammatory stress, confirming the biocompatibility of the nanoformulations. However, both LN+APAP and LMN+APAP attenuated inflammatory cytokines induced by APAP, with LMN+APAP showing slightly stronger reductions in TNF-α by 29% and IL-1β by 21%, probably as a result of the following: (i) lignin inherent antioxidant phenolic structures capable of neutralizing reactive intermediates and dampening inflammatory signaling, (ii) enhanced bioavailability of the encapsulated bioactive substance, and (iii) liver targeting. The group M+APAP displayed partial normalization of the hepatic cytokine balance: 27% decrease in IL-6 and 38% increase in TNF-α vs. these of the APAP group, suggesting that, although the bioflavonoid possesses potent antioxidant and anti-inflammatory properties, its limited solubility and poor hepatic bioavailability are probably responsible for its reduced in vivo activity.

In general, the co-treatment with LMN and APAP provoked the most pronounced normalization of cytokine levels, with marked reductions in IL-6, TNF-α, and IL-1β and near-control IL-10 values. The latter conclusion indicates that encapsulating morin within lignin nanoparticles greatly enhances its stability, solubility, and sustained delivery to hepatic tissue, thereby potentiating its anti-inflammatory activity. Overall, LMN act as an efficient carrier that enhances morin hepatoprotective activity, suppresses the inflammatory cascade triggered by APAP toxicity, and restores the liver cytokine equilibrium more effectively than morin or LN alone. The latter results were supported by the analyses of the statistical significance of the experimental data presented in the Tukey post hoc *p*-value matrix for IL-10, IL-6, TNF–α, and IL-1β in Table 2.

The modern scientific literature presents studies providing evidences on the potential of the natural polyphenolic biopolymer, lignin, and the natural flavonoid morin in reducing inflammation by inhibiting the production of cytokines and interleukins [47,48]. Kamal et al. proved the antioxidant and anti-inflammatory mechanism of morin in the amelioration of paracetamol-induced toxicity in rats. The scientific team observed that the administration of the flavonoid depleted the expression of nuclear factor-κβ (NF-κB), NADPH oxidase 2 (NOX-2), and IL-6 and alleviated the significant increase in the OH-1 level compared with paracetamol administration only [49]. Kumar et al. established that morin hydrate ameliorates di-2-ethylhexyl phthalate (DEHP)-induced hepatotoxicity in mice via tumor necrosis factor alpha (TNF-α) and NF-κβ signaling [50]. The liver MDA level was significantly increased with a concomitant decrease in enzymic antioxidant activities in SV-administered rats. SV overdose also caused the upregulation of pro-inflammatory markers in liver tissue, presence of inflammatory cell infiltration, and hepatocellular necrosis [51]. Mondal et al. obtained results similar to ours, indicating that the co-treatment of morin, vitamin E, and morin-encapsulated chitosan nanoparticles or morin-vitamin E-β-cyclodextrin inclusion complex-loaded chitosan nanoparticles with arsenic induced hepatotoxicity in mice and reduced liver function markers, ROS levels, pro-apoptotic and inflammatory factors with an elevation of antioxidant parameters and improvement of liver histopathology. Their studies provided evidence that the effect of the treatment with both types of morin-loaded nanoparticles was significantly improved as compared to these of morin alone [41,52].

The observed enhancement of the hepatoprotective and anti-inflammatory behavior of flavonoid-encapsulated biopolymer nanoparticles in vivo is strongly supported by the following evidences. Firstly, according to previous studies, liver tissue can absorb particles up to 250 nm in size, and better internalization was proven by treatment with spherical nanoparticles [53,54]. Our previously published study substantiated these findings as it reports spherical or oval shapes of the applied in the current investigations of LN and LMN and sizes within the range 50–100 nm [18]. Secondly, the bioflavonoid-loaded polymeric nanoparticles display higher water solubility and sustained release of the incorporated bioactive compounds [19,55]. And on the third place, the conjugated nanoformulations exhibit enhanced bioavailability, improved biodistribution, ameliorated bioactivities including enhanced antioxidant activity [20,56], and improved antiproliferative and antibacterial potential [57] as compared to the pure substance/s [58,59].

The correlation analysis of the experimental data (Table 3) reveals a complex interplay between oxidative stress, antioxidant defenses, inflammatory cytokines, and liver function markers.

MDA, a key indicator of lipid peroxidation, is strongly positively correlated with CAT (r = 0.844), liver enzymes AST (r = 0.659), ALT (r = 0.948), and ALP (r = 0.742), as well as with the pro-inflammatory cytokine IL-6 (r = 0.779), highlighting a close association between oxidative damage, inflammation, and hepatocellular injury. Conversely, MDA is negatively correlated with SOD (r = −0.317) and GPx (r = −0.754), suggesting that higher antioxidant enzyme activity could be associated with reduced oxidative stress. SOD and GPx themselves show strong negative correlations with inflammatory markers IL-6 (r = −0.787 and −0.904, respectively), IL-10 (r = −0.817 and −0.636), TNF-α (r = −0.708 and −0.294), and IL-1β (r = −0.638 and −0.317), reinforcing their role in counteracting both oxidative and inflammatory responses. Interestingly, IL-10, although anti-inflammatory, is positively correlated with the pro-inflammatory cytokines TNF-α (r = 0.857) and IL-1β (r = 0.741), which may reflect a compensatory anti-inflammatory response during heightened inflammation. Liver enzymes are consistently positively correlated with MDA, CAT, and inflammatory cytokines, indicating that oxidative stress and inflammation are closely linked to hepatic dysfunction.

Collectively, despite of the limited database, these relationships imply a notable feedback network where oxidative stress promotes inflammatory cytokine release and hepatocellular damage, while endogenous antioxidant and anti-inflammatory responses attempt to restore homeostasis. This coordinated interplay underscores the critical balance between oxidative damage, inflammation, and liver function in pathological conditions.

Importantly, the key trends, including the greater hepatoprotective efficacy of LMN compared with LN or morin alone, were reproduced consistently in each of the two repeats. Averaging the datasets provided a representative summary of the reproducible effects observed across experiments. This approach strengthens confidence in the robustness of the findings, particularly for biochemical and cytokine endpoints that can exhibit inter-experiment variability, and supports the conclusion that LMN exerts a reliably superior protective effect in the APAP injury model.

#### 2.3.4. Effect of the Bioflavonoid and Nanoparticles on Hepatic Histopathological Changes

The present study investigates the hepatoprotective potential of LN, M, and LMN in a model of experimentally induced APAP hepatotoxicity, with histopathological evaluation serving as the principal criterion for assessing liver tissue injury.

Liver sections were evaluated using an ordinal semi-quantitative scoring system with whole numbers from 0 to 4, where 0 represents normal tissue and 4 represents the maximum severity of the feature. A 5-level scale (0–4) was chosen to balance sensitivity to detect group differences with repeatability [60]. Most descriptors were assessed qualitatively by trained operators based on descriptive criteria. All histological evaluations were performed by two independent observers blinded to group allocation, and inter-observer reliability was confirmed (Cohen’s κ > 0.85). The scoring criteria for each descriptor are summarized in Table 4.

**Figure 5:**
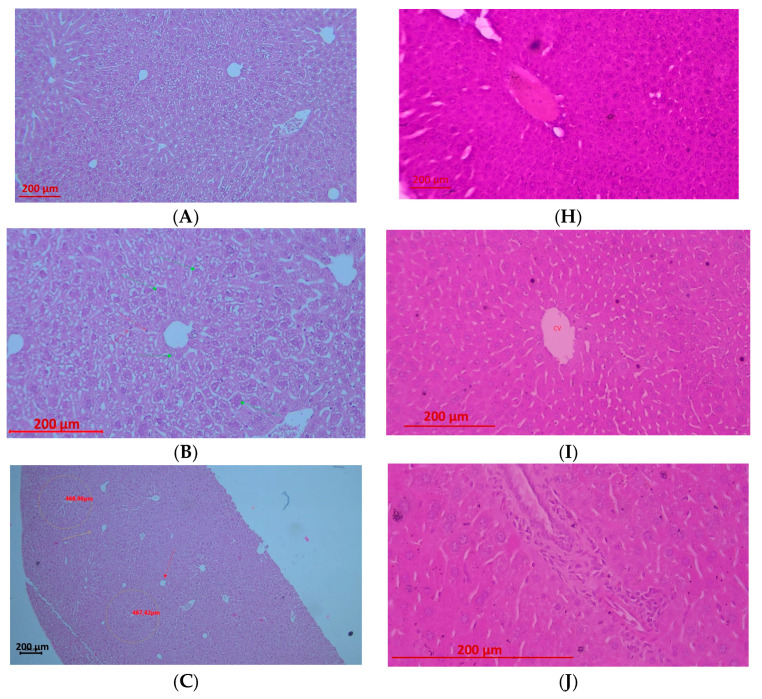

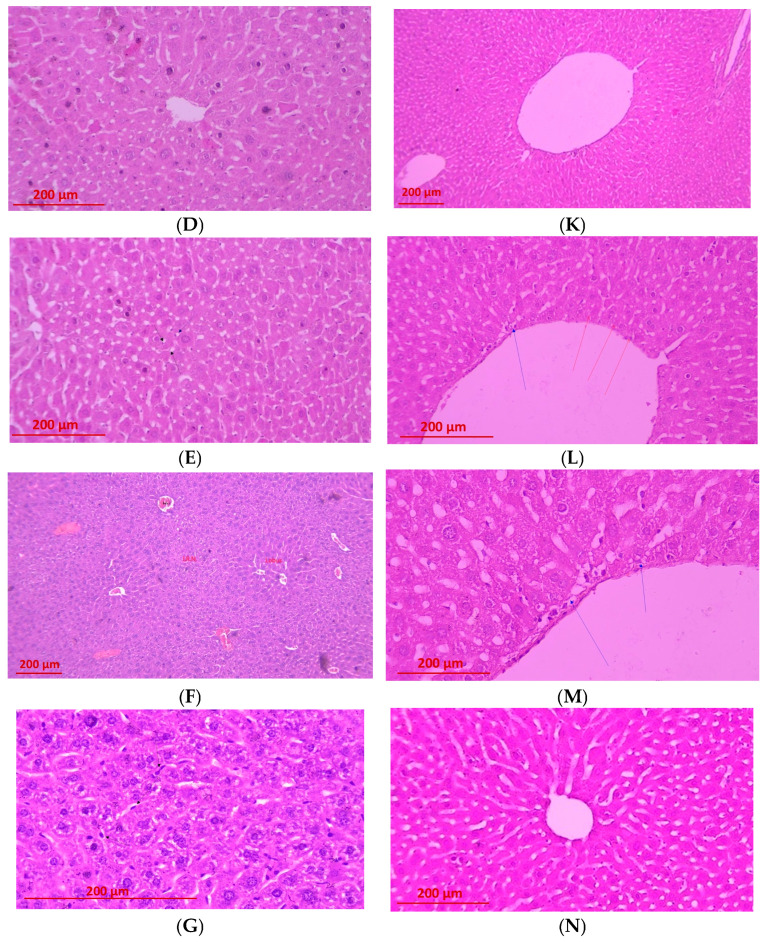
Effect of M, LN, and LMN on the hepatic histopathological changes of APAP-induced liver injury in mice: (**A**) APAP group: necrotically altered areas, which are not visible macroscopically, but in the cytoplasm of some cells, fatty infiltration is present, and in others, total obliteration of the structure is visible; (**B**) APAP group: green arrows indicate necrosis, karyorrhexis, and karyolysis and red arrows—activation of Kupffer cells (magnification 20×); (**C**) APAP group: diffusely scattered microscopic changes displaying dilated central veins and interstitial reaction (yellow arrow) and the borders of the lobes (yellow circles) (magnification 5×); (**D**) APAP group: numerous empty spaces with cells that are punctate or slightly elongated in shape, giving information about fatty infiltration of the organ (magnification 20×); (**E**) APAP group: adipocytes (black arrows); (**F**) LN group: compressed lobes of the interstitial reaction and necrosis (magnification 10×); (**G**) LN group: a section of the interstitium, in which Kupffer cells are activated (black arrows), some of which are in the process of phagocytosis (magnification 40×); (**H**) M group: hyperemia of V. centralis and preserved liver structure (magnification 10×); (**I**) LM group: edema and dilation of V. centralis (magnification 20×); (**J**) LM group: lymphocyte—mesenchymal reaction and stenosis of the coronary vessel (magnification 40×); (**K**) LN+APAP group: fatty infiltration of the liver tissue (magnification 10×); (**L**) LN+APAP group: red arrows that indicate necrobiotic changes and blue arrow—fatty degeneration (magnification 20×); (**M**) LN+APAP group: background that indicates the empty spaces of fatty degeneration, peripherally of which there are degenerative changes and blue arrows—necrobiotic changes at the base of V. centralis; (**N**) LMN+APAP: highly edematous tissue with stagnant processes without evidence of necrosis and degenerative changes.

The morphological alterations observed across the experimental groups clearly demonstrate differing degrees of hepatic protection and align with the established mechanisms of APAP toxicity and the biological activities of the tested compounds and formulations (Figure 5).

Semi-quantitative histopathological scoring revealed severe hepatic injury in the APAP group, characterized by high necrosis, pronounced inflammatory infiltration, and extensive vacuolization (scores 3–4). LN alone produced mild to moderate alterations, including necrosis (1–2), moderate inflammation (2), and minimal vacuolization (1–2). Morin (M) treatment markedly reduced tissue damage, showing minimal necrosis (0–1) with mild inflammation and vacuolization (score 1). LMN alone exhibited negligible necrosis (0–1), mild inflammatory changes (1), and mild vacuolization (1), with vascular edema as the predominant finding. In APAP-challenged groups, LN+APAP displayed moderate injury with necrosis, inflammation, and vacuolization ranging from 2 to 3, whereas LMN+APAP demonstrated the greatest hepatoprotection, with absence of necrosis (0), minimal inflammation (1), and minimal vacuolization (0–1), limited to mild reversible vascular changes.

The APAP-treated group exhibited the characteristic centrilobular necrosis associated with toxic metabolite accumulation, specifically NAPQI. Pronounced karyolysis, karyopyknosis, and karyorrhexis, together with diffusely distributed necrobiotic changes, substantiate the severity of the insult (Figure 5A–E). Concurrent fatty infiltration reflects underlying metabolic derangement and mitochondrial dysfunction. These findings are fully consistent with the classical pathological paradigm of APAP-induced hepatic injury described in the literature and provide a robust reference point for evaluating the protective potential of the remaining treatments.

The administration of LN alone did not elicit severe parenchymal destruction; however, the nanoformulation induced a marked interstitial reaction characterized by hyperemia, Kupffer cell activation, and necrobiotic changes confined to the interstitium (Figure 5F,G). Preservation of the centrilobular architecture suggests a moderate toxic-irritative effect, potentially attributable to metabolic burden or interactions with immunocompetent cells. Moreover, LN did not exert hepatoprotective activity in the context of APAP toxicity, as evidenced in the LN+APAP group (Figure 5K,M), where fatty degeneration and necrobiotic alterations remained. This partially reflects findings by Orlov et al., where lignin-derived polyphenols exert antioxidant and immunomodulatory effects, suggesting that lignin may modulate immune responses while providing moderate radical scavenging [35]. The bioflavonoid morin exerted a distinct hepatoprotective effect, with histological changes limited to mild edema and pronounced dilatation of the central vein in the absence of necrosis or significant degenerative lesions. This outcome is concordant with the well-documented antioxidant properties of morin, including attenuation of oxidative stress, stabilization of cellular membranes, and modulation of inflammatory signaling pathways, which likely underpin its protective action against NAPQI-mediated injury [61]. The LMN group characterized with substantial reduction in hepatic injury, with the predominant changes being hemodynamic in nature—edema within the spaces of Disse, moderate disruption of hepatic plates, and marked dilatation of centrilobular veins. The presence of minimal degenerative alterations indicates that morin effectively counteracts the inflammatory response elicited by lignin. These findings suggest a possible additive interaction, likely driven by the combined antioxidant capacities of both bioactive components, yet also imply that lignin alone may partially attenuate the full expression of the protective effects of morin.

In certain specimens on the LN+APAP group fatty degeneration appeared accentuated, potentially reflecting interactions between lignin and lipid metabolism or altered phagocytic activity of Kupffer cells. The most notable outcome was observed in the LMN+APAP group, in which neither necrosis nor substantive degenerative changes were detected. The histological appearance was dominated solely by edema and vascular congestion—lesions that are considered mild and reversible. The latter findings indicate a pronounced additive effect between lignin and morin, wherein morin provides potent antioxidant defense while lignin may function as a delivery vehicle, radical-scavenging agent, or modulator of local immune activity. The study of Brzhozovskiy et al. investigated several multicomponent natural mixtures, including a lignin derivative in a mouse model of subacute liver failure induced by CCl_4_. Proteomic and metabolomics research to examine how the addition of activated hydrolytic lignin and humic acid peloids affected metabolic pathways in mice with steatosis were performed. Both complex mixtures showed moderate in vitro and weak in vivo activity, but their impact on the composition of the proteome and the biochemical reactions in mice was noticeable [62].

In conclusion, the histopathological evidence supports the conclusion that morin exhibits remarkable hepatoprotective properties. Besides, its encapsulation into LN significantly enhances this protective activity even under conditions of severe APAP-induced hepatic injury.

### 2.4. Proposed Hepatoproetective Mechanism of LN and LMN Against APAP-Induced Liver Injury

**Figure 6:**
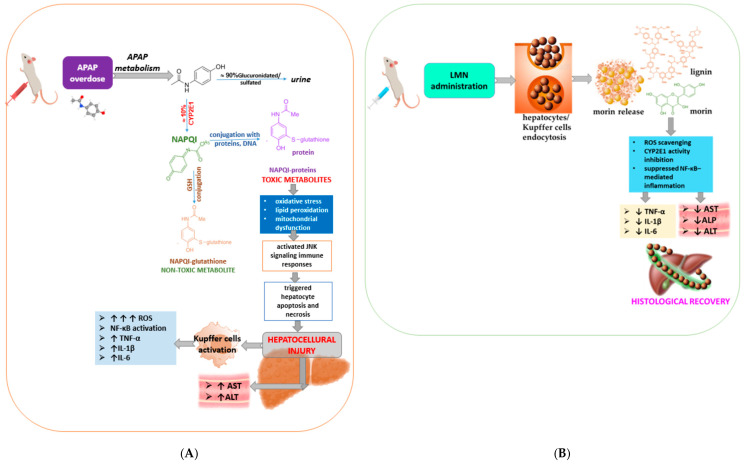
Mechanistic scheme showing the probable mechanism of morin-encapsulated lignin nanoparticles’ mitigation of APAP-induced liver injury: (**A**) molecular mechanism of APAP-induced hepatotoxicity and (**B**) molecular mechanism of alleviation of APAP-induced liver injury via treatment with LMN. Adapted from [15,66,67,68].

Overall, the experimental data presented clearly demonstrate that APAP induces severe hepatic injury, while morin, LN, and especially LMN mitigate this damage to varying extent. LMN provide a pronounced hepatoprotective effect, normalizing liver enzyme levels close to those of the control group, indicating that morin-encapsulated lignin nanoparticles represent a superior formulation for protection against oxidative liver injury. This formulation combines the natural antioxidant potency of morin with the delivery efficiency and biocompatibility of lignin nanoparticles, highlighting their potential as a promising protective strategy for managing drug-induced hepatotoxicity. The proposed mechanism of morin-encapsulated lignin nanoparticles’ mitigation of APAP-induced liver injury in mice is schematically presented in Figure 6.

Acetaminophen (paracetamol, 4-hydroxyacetanilide, N-acetyl-para-aminophenol, or APAP) is a pain reliever for acute and chronic pain that can be purchased over-the-counter (OTC) [63]. At physiological pH, it is almost completely neutral and is absorbed from the duodenum. Its elimination occurs in the liver, where most of it is glucuronidated or sulfated and then excreted in the urine. A smaller portion is oxidized by cytochrome P450 to 3-hydroxy-APAP and N-acetyl-p-benzoquinone imine (NAPQI). NAPQI is usually not harmful, as it binds to glutathione in the liver and is excreted in the bile [64]. Excessive intake of acetaminophen (APAP) leads to its bioactivation by hepatic CYP2E1—a liver enzyme involved in the metabolism of various substances, including alcohol, drugs, and fatty acids, into the toxic metabolite NAPQI (Figure 5A). When glutathione (GSH) stores are exhausted, NAPQI binds covalently to cellular proteins, producing oxidative stress, lipid peroxidation, and mitochondrial dysfunction, which activate JNK (c-Jun N-terminal kinase, a crucial cellular pathway that responds to stress and regulates cell growth, differentiation, and apoptosis) signaling and immune responses and trigger hepatocyte apoptosis and necrosis [65]. Damaged hepatocytes release damage-associated molecular patterns that activate Kupffer cells, a key event in liver injury, as activated Kupffer cells release pro-inflammatory cytokines and reactive oxygen species (ROS) and also recruit other immune cells to the site, leading to NF-κB activation and elevated pro-inflammatory cytokines (TNF-α, IL-1β, and IL-6).

To counteract this cascade, newly engineered LMN were administered, in which lignin serves as a biocompatible scaffold encapsulating morin, a flavonoid with potent antioxidant and anti-inflammatory properties. These nanoparticles possess enhanced controlled-release capacity [32,69], allowing for efficient hepatic targeting and cellular uptake via endocytosis by hepatocytes and Kupffer cells (Figure 6B). Once internalized, LMN gradually release morin, which directly scavenges ROS. Simultaneously, morin causes inhibition of CYP2E1 activity, suppressing of NF-κB-mediated inflammation provoking stabilization of mitochondrial membranes. The additive effects of morin and the bioactive heteropolymer play a significant role in restoring the redox balance, maintaining mitochondrial integrity, and preventing hepatocyte death. Consequently, LMN treatment significantly lowers oxidative stress and inflammation, leading to reduced serum AST, ALT, and ALP levels and preservation of hepatic histoarchitecture.

**Figure 7:**
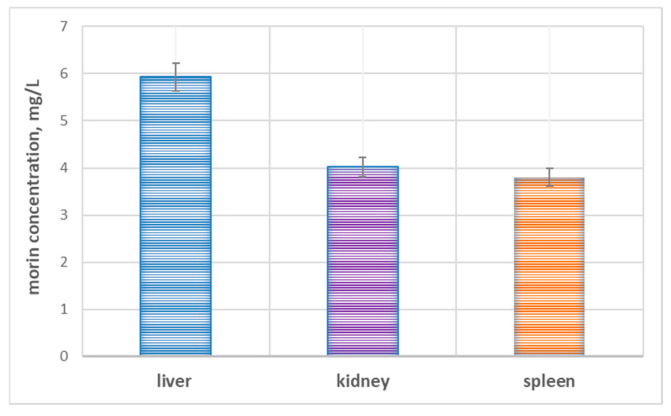
Biodistribution of morin in liver, kidney, and spleen homogenates in mice treated with LMN in a dose of 50 mg/kg.

It is important to note that the analysis of the biodistribution of morin after administration of LMN (50 mg/kg) further supports the hepatotropic behavior of the nanoparticles. The highest concentration of morin was found in the liver (5.93 ± 0.295 mg/L), followed by lower levels in the kidney (4.02 ± 0.201 mg/L) and spleen (3.80 ± 0.156 mg/L). The observed distribution pattern (Figure 7), substantiated by other scientific teams [70] indicates preferential accumulation of LMN in the liver tissue, which is consistent with the intended therapeutic target.

The increased concentration in the liver confirms the efficient delivery and retention of morin at the site of APAP-induced injury, allowing for prolonged antioxidant and anti-inflammatory activity. Meanwhile, the moderate levels found in the kidney and spleen likely reflect the physiological clearance and uptake by the reticuloendothelial system, respectively, which is typical of lignin-based nanocarriers. Taken together, these biodistribution data confirm that the enhanced hepatoprotective efficacy of LMN stems not only from the intrinsic bioactivity of morin, but also from the targeted and efficient delivery provided by the conjugated nanoparticle formulation. Across multiple nanoparticle platforms (chitosan, poly lactic-co-glycolic acid or PLGA, mesoporous silica, and self-nanoemulsifying drug delivery system), liver consistently shows the highest morin levels, with kidney and spleen usually lower but detectable [71,72,73].

Compared with previously reported studies on flavonoid-loaded nanoparticles (Table 5), LMN provide a distinct combination of attributes that strengthen their therapeutic relevance in APAP hepatotoxicity. Polymeric carriers such as chitosan, alginate, or PLGA primarily function as inert delivery vehicles that enhance flavonoid stability or control release but contribute little intrinsic antioxidant activity. Lipid-based nanoemulsions and self-nanoemulsifying drug delivery systems improve oral absorption but lack organ targeting and rely entirely on the encapsulated flavonoid for protection. In contrast, LMN integrate morin with a lignin matrix that possesses its own polyphenolic, ROS-scavenging properties, creating a dual-active system capable of augmenting redox buffering at the site of injury. Additionally, the natural tendency of lignin nanoparticles to accumulate in liver and spleen via RES uptake supports more efficient hepatic delivery than many lipid systems. This combination of bioactive carrier, renewable material, and hepatotropic accumulation differentiates LMN from existing flavonoid nanoplatforms and emphasizes the novelty of the presented study. Besides, nanoparticles below 100 nm typically show higher cellular internalization and more advantageous biodistribution patterns, enabling more efficient delivery to liver tissues. Consequently, the hepatoprotective efficacy seen in our study may be partly attributed to this optimized size range, which promotes more efficient intracellular transport and sustained local bioavailability.

### 2.5. Limitations of the Study

In the present study, LMN administration significantly attenuated APAP-induced hepatotoxicity, as evidenced by reductions in serum transaminases, decreased oxidative stress markers, and improved histopathological scores. These findings indicate that LMN effectively mitigates hepatic injury under the experimental conditions used. However, the underlying molecular mechanisms cannot be fully defined based on the current dataset. Despite the promising hepatoprotective effects of morin, lignin nanoparticles (LN), and morin-loaded lignin nanoparticles (LMN) against acetaminophen-induced hepatotoxicity, several limitations should be acknowledged.

First, the study was conducted solely in mice, which may not fully represent human hepatic physiology or immune responses. The work also focused on acute liver injury, without assessing long-term safety, chronic exposure, or repeated dosing effects.

Furthermore, dose optimization and pharmacokinetic evaluations were not performed, limiting understanding of the optimal therapeutic range and systemic distribution of LMN. Although lignin-based nanocarriers and morin have been reported elsewhere to influence pathways such as oxidative stress modulation, inflammatory signaling, and mitochondrial integrity, these mechanisms were not directly evaluated here. Accordingly, our conclusions are limited to the observed biochemical and morphological outcomes, and future studies incorporating targeted molecular assays will be necessary to elucidate the specific pathways through which LMN exert hepatoprotection.

Additionally, as well-documented sex-specific differences exist in APAP metabolism, redox responses, and susceptibility to liver injury, the use of only male mice represents a limitation of this study. Consequently, the generalizability of our findings is restricted, and future investigations incorporating both sexes will be necessary to determine whether the hepatoprotective effects of LMN extend broadly across biological sex.

Future studies should also address these limitations through extended mechanistic, pharmacokinetic, and translational investigations to validate LMN as a potential hepatoprotective nanotherapeutic.

## 3. Materials and Methods

### 3.1. Synthesis and Characterization of Lignin and Morin-Encapsulated Lignin Nanoparticles

#### 3.1.1. Synthesis Methodology of the Nanoparticles

LN and LMN were synthesized by an ethanol antisolvent method, proposed by Parlapanska et al. [18] and Yaneva et al. [69]. In brief, lignin nanoparticles (LN) were prepared by dissolving alkali lignin (125 mg in 25 mL ultrapure water at 5 mg/mL), adding 1 mL ethanol, stirring at 500 rpm for 3 min, then inducing precipitation by dropwise addition of 7 mL of 1% citric acid (~4 mL/min) with continued stirring for 10 min. The resulting suspension was centrifuged (15,000× *g* at 10 °C for 30 min), washed three times, ultrasonicated on ice (96% intensity), and lyophilized at −64 °C. The lyophilized lignin nanoparticles (LNPs) and morin-encapsulated lignin nanoparticles (LMN) were stored in airtight containers at 4 °C until use, protected from light and moisture. For morin-loaded nanoparticles morin (0.04 g in 1 mL EtOH) was added prior to precipitation followed by identical steps cited for the blank LN.

#### 3.1.2. Physicochemical, Morphological, and Spectroscopic Characterization of the Nanoparticles

The FTIR spectra of LN and LMN within the wavelength range 400–4000 cm^−1^ were obtained by the KBr disc technique on a TENSOR 37 Bruker FTIR spectrometer (Bruker Optik GmbH, Ettlingen, Germany).

For TEM analysis, the nanoparticle suspensions were stained with 1% uranyl acetate in 70% methanol and deposited onto electron microscopy grids pre-coated with a thin formvar film. TEM imaging was conducted at high resolution on a HR-STEM JEOL JEM-2100 transmission electron microscope (JEOL Ltd., Tokyo, Japan), equipped with a GATAN Orius 832 SC1000 CCD camera (Gatan GmbH, Munich, Germany).

The structure of the nanoparticles was characterized by X-ray diffraction (XRD) instrument (PANalytical Empyrean CuKα = 0.15406 nm, 40 kV, and 40 mA) (Malvern Panalytical, Spectris Company, London, UK) in a 2θ range from 4° to 70°.

The ζ-potential and particle size of the nanoparticles were measured using a Malvern particle analyzer (Malvern Panalytical, Spectris Company, London, UK) at 25 °C. Each measurement was performed in triplicate to ensure reproducibility.

#### 3.1.3. In Vitro Release Study

Experiments assessing the in vitro release kinetics of the flavonoid morin from LN were conducted in a glass batch reactor equipped with a mechanical stirrer. The morin-loaded nanoparticles were dispersed in simulated gastrointestinal media (free of enzymes) at pH 1.2, 7.4, and 6.8, under a constant temperature T = 37 ± 0.5 °C in a WNB 22 digital water bath (Memmert GmbH, Schwabach, Germany). Samples were collected at predetermined time intervals, and morin concentration in the liquid phase was quantified spectrophotometrically at λ = 390 nm. To prevent saturation of the remaining solution, the withdrawn volume was replaced with an equal volume of fresh medium. All experiments were performed in triplicate, and mean values were used to minimize random error. Morin-free blanks and replicate samples at each time point were included in all experimental runs.

### 3.2. Ethical Approval

The experiment was conducted in accordance with the requirements of Regulation No. 20/01.11.2012 of the minimum requirements for the protection and welfare of experimental animals and the sites used for their breeding, use, or delivery [61]. The experimental setup was approved by the Animal Ethics Commission of the Faculty of Veterinary Medicine, Trakia University, Stara Zagora, Bulgaria and the Bulgarian Food Safety Agency, Sofia, Bulgaria with a License No. 390/6000-0334/18 April 2024.

### 3.3. Experimental Animals

The object of the study were white mice Mus musculus albus, ICR line, delivered for the needs of the experiment from the vivarium of the Medical University, Plovdiv. All in vivo experiments were performed twice independently with six animals per group, and the experimental data presented in the current study represent the averaged outcomes across both experimental repeats. A total of 48 male specimens aged 2 months with an average weight of 36.92 g ± 3.69 were selected and divided into 8 groups of 6 individuals for each of the two independent experiments. Only male mice were included in this study to reduce variability and maintain consistency with previous studies of APAP-induced hepatotoxicity. We acknowledge that sex-specific differences in susceptibility to APAP and in hepatic redox and inflammatory pathways have been documented, with females sometimes showing lower susceptibility due to estrogen-mediated hepatoprotection [81]. Therefore, the findings of the present study are limited to male mice, and future studies including both sexes are warranted to assess whether LMN efficacy, biodistribution, and hepatoprotective mechanisms differ between males and females.

### 3.4. Experimental Conditions

Animals were housed in the vivarium of the Faculty of Medicine, Trakia University, Stara Zagora, Bulgaria. The animals had a 10-day cycle of adaptive feeding and acclimatization. During the experiment, they had free access to fresh water and food and were maintained in 12 h/d light/dark cycles, 23 ± 2 °C, and a humidity at 55%.

### 3.5. Experimental Procedure

The experimental animals were randomly assigned to eight groups (*n* = 6 per group) (Table 6) using a simple randomization procedure before treatment. Group allocation was performed by an investigator not involved in subsequent experimental procedures to maintain allocation concealment. All experiments were independently repeated twice, with six animals per group (*n* = 6). All biochemical and histopathological analyses were conducted by researchers blinded to group assignments. Furthermore, personnel responsible for animal care and sample collection were distinct from those performing data analysis, ensuring compliance with ARRIVE guidelines for randomization and blinding. Group 1 (control) received intraperitoneal injections of saline for three consecutive days. Group 2 received acetaminophen (200 mg/kg, i.p.) for three consecutive days. Group 3 received morin (50 mg/kg, i.p.) for five consecutive days. Group 4 received lignin nanoparticles (50 mg/kg, i.p.) for five consecutive days. Group 5 received a suspension of lignin/morin nanoparticles (50 mg/kg, i.p.) for five consecutive days. Group 6 received morin (50 mg/kg, i.p.) for five consecutive days, followed by acetaminophen (200 mg/kg, i.p.) for three consecutive days. Group 7 received lignin nanoparticles (50 mg/kg, i.p.) for five consecutive days, followed by acetaminophen (200 mg/kg, i.p.) for three consecutive days. Group 8 received a suspension of lignin/morin nanoparticles (50 mg/kg, i.p.) for five consecutive days, followed by acetaminophen (200 mg/kg, i.p.) for three consecutive days.

The clinical status and behavior of the animals were monitored daily. Blood samples were collected by intracardiac puncture using vacuum serum tubes after induction of anesthesia with 100% isoflurane. Samples were centrifuged at 4000 rpm for 10 min. Animals were euthanized by cervical dislocation in accordance with Regulation No. 20/01.11.2012 of the minimum requirements for the protection and welfare of experimental animals and the sites used for their breeding, use, or delivery [82].

### 3.6. Biochemical Analyses

Animals were euthanized 24 h after the final injection. Blood was collected by cardiac puncture, and serum was separated for biochemical assays. Liver tissues were excised immediately for oxidative stress, cytokine, and histopathological analyses.

#### 3.6.1. Functional Markers of Liver Injury

Serum concentrations of aspartate aminotransferase (AST), alanine aminotransferase (ALT), and alkaline phosphatase (ALP) were quantified using an automated biochemical analyzer (Mindray BS-120, Shenzhen, China) with commercial assay kits (Biolabo S.A.S., Maizy, France). All measurements were conducted at the Clinical Laboratory of the Laboratory and Diagnostic Center, Trakia University—Stara Zagora, Bulgaria.

#### 3.6.2. Pro-Inflammatory Cytokine Quantification

Serum levels of IL-1β, IL-6, IL-10, and TNF-α were measured using enzyme-linked immunosorbent assay (ELISA) kits: Biomatik Mouse IL-1β ELISA Kit (Cat. EKF57763, sensitivity 7.5 pg/mL), Biomatik Mouse IL-6 ELISA Kit (Cat. EKF57767, sensitivity 9.375 pg/mL), Biomatik Mouse IL-10 ELISA Kit (Cat. EKC40065, sensitivity 15.6 pg/mL), and Biomatik Mouse TNF-α ELISA Kit (Cat. EKF57781, sensitivity 2.344 pg/mL), respectively, following the manufacturers’ protocols.

#### 3.6.3. Antioxidant Enzyme Activity in Liver Tissue

The activity of glutathione peroxidase (GPx) in liver homogenates was determined using an ELISA-based detection method according to the instructions supplied with the commercial kit (Cat. ELK7785, sensitivity 0.59 ng/mL).

#### 3.6.4. Spectrophotometric Methods for Oxidative Stress Evaluation

Modified spectrophotometric protocols were employed to assess oxidative stress markers in liver tissue homogenates. The biochemical analyses were performed on a UV–VIS spectrophotometer DR 4000 (Hach, Berlin, Germany)

The liver cellular activity of superoxide dismutase (SOD), catalase (CAT), malondialdehyde concentration (MDA) were estimated by modified protocols based on the methods of Sun et al., Aebi, Plaser et al., and Karamalakova et al. [83,84,85,86].

### 3.7. Histological Analysis

Liver samples were collected within 6 min of post-cardiac arrest to minimize autolysis. Tissues were fixed in 10% buffered formalin for 24 h, rinsed with distilled water, and re-fixed to ensure optimal preservation. Fixed tissues were trimmed for embedding using a Leica Histo Pearl tissue processor (Leica Biosystems, Nussloch, Germany) with a 24 h stepwise protocol. Samples included whole organs or representative sections selected based on anatomical landmarks and pathological changes. Paraffin embedding was performed using a grossing station (Myr EC 500-1) with paraffin (melting point 45 °C) (Merck, Rahway, NJ, USA). Paraffin blocks were sectioned on a semi-automatic microtome (Myr M-240) to obtain longitudinal and cross-sectional slices. Histological sections were stained with Hematoxylin-Eosin (Merck) using an automated stainer (Leica ST4020, Leica Biosystems, Nussloch, Germany). Stained slides were examined with a Kern OBN 135 optical micro-scope (Kern & Sohn, Balingen, Germany) equipped with an Euromex VC.3034 camera. Cover slip-ping was performed with liquid mounting medium (Merck, Rahway, NJ, USA).

### 3.8. Reagents

The following reagents were used in the biochemical and spectrophotometric assays: Paracetamol (B. Braun, Melsungen, Germany); NaCl 0.9% (B. Braun, Melsungen, Germany); Lignin (alkali, CAS No. 8068-05-1); morin (CAS No. 654055-01-3); DPPH (CAS No. 1898-66-4); ABTS™ chromophore (CAS No. 30931-67-0); ethanol (≥99.8%); phosphate-buffered saline (PBS, P-3813); phosphoric acid (CAS No. 7664-38-2); trichloroacetic acid (CAS No. 76-03-9); thiobarbituric acid (CAS No. 504-17-6); chloroform/ethanol mixture (CAS No. 67-66-3); Tris-HCl buffer; HCl (CAS No. 7647-01-0); and pyrogallol (CAS No. 87-66-1). All reagents were purchased from Sigma-Aldrich (St. Louis, MO, USA) unless otherwise specified.

### 3.9. Spectrophotometric Determination of Morin in Homogenates

**Figure 8:**
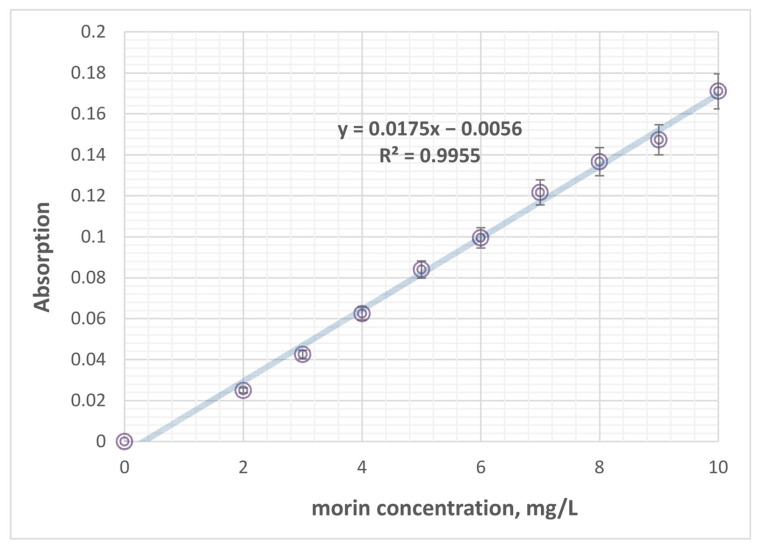
Calibration curve of morin within the initial concentration interval of 2.0–10 mg/L.

Morin concentrations in tissue samples were quantified using a modified, by us, spectrophotometric method based on the methodology of [87] the formation of a morin–AlCl_3_ complex with a characteristic absorption maximum at 416 nm. Briefly, weighed tissue samples were homogenized in 2 mL acetonitrile and vortexed for 4–5 min to ensure complete extraction. The homogenates were then centrifuged at 10,000 rpm for 10 min at 4 °C, followed by an additional 10-min incubation at 4 °C to facilitate phase separation. A volume of 1100 µL of the resulting supernatant was carefully collected and extracted with 1.6 mL absolute ethanol (EtOH). Prior to measurement, the spectrophotometer was zeroed against absolute ethanol. The combined extract (supernatant + EtOH) was mixed with a 3 mL AlCl_3_ solution (100 mg/L) to allow complex formation. After homogenization and a short stabilization period (1–2 min), the absorbance of the morin–AlCl_3_ complex was recorded at λ = 416 nm. Morin concentrations in samples were calculated using a calibration curve (Figure 8) prepared under identical chemical conditions.

### 3.10. Statistical Analysis

Statistical analyses were performed using XLSTAT version 2021.5 statistical software for Excel (Microsoft Corporation, Redmond, WA, USA). Numerical data were expressed as mean ± standard deviation (±SD) and presented graphically. Differences between and within the experimental groups were determined using a one-way analysis of variance (ANOVA) followed by post hoc Tukey tests to correct for multiple comparisons. *p*-Values < 0.05 were indicative of statistical significance.

## 4. Conclusions

The oxidative stress data revealed that APAP exposure drastically disrupts hepatic redox homeostasis in mice with induced hepatotoxicity, as evidenced by the elevated MDA levels and suppressed SOD and GPx activities. Treatment with morin, LN, and LMN nanoparticles effectively counteracted these effects, restoring the antioxidant defenses to varying degrees. LN exhibited strong intrinsic antioxidant activity, significantly lowering lipid peroxidation and normalizing SOD, CAT, and GPx levels. Morin alone also attenuated oxidative damage but less effectively, likely due to limited solubility and bioavailability. The morin-loaded lignin nanoparticles provided comparable protection, reducing MDA and enhancing enzymatic activities, indicating that the nanoparticle system offers sustained morin release and enhanced protection. The results suggest that both Lignin nanoparticles and LMN formulations confer significant hepatoprotection through free radical scavenging, enzyme stabilization, and reinforcement of the glutathione-dependent defense system, making them promising therapeutic agents against APAP-induced oxidative liver injury. The study demonstrated that APAP administration induced significant hepatic inflammation, as evidenced by the elevated levels of pro-inflammatory cytokines (IL-6, TNF-α, and IL-1β) and compensatory IL-10 increase. Treatment with LN or LMN alone showed no inflammatory response, confirming their safety and biocompatibility. Co-treatment with LMN and APAP most effectively restored cytokine balance, significantly reducing IL-6, TNF-α, and IL-1β while normalizing IL-10 levels. This superior anti-inflammatory and hepatoprotective effect of LMN over morin or lignin alone results from morin encapsulation within lignin nanoparticles, which enhances its solubility, stability, bioavailability, and targeted hepatic delivery.

The present study highlights significant advancements in the domain of hepatoprotection, emphasizing the improved potential of natural compounds integrated with biopolymer-based nanotechnology. These findings contribute to the evolving framework for the development of next-generation therapeutic strategies aimed at preventing and treating liver disorders.

## Figures and Tables

**Table 1:** Average size, ξ-potential, and pH of blank and quercetin-loaded lignin/chitosan nanoparticles and their suspensions.

Nanoparticle Type	Size, nm	ξ-Potential, mV	pH
LN	50.6 ± 2.02–82.5 ± 4.12	−30.13 ± 1.21	9.65
LMN	59.8 ± 2.87–89.7 ± 4.75	−33.68 ± 1.68	8.97

**Table 2:** Tukey post hoc *p*-value matrix for experimentally measured hepatic inflammatory cytokines.

IL-10, pg/mL								
	control	APAP	LMN	LN	LN+APAP	M+APAP	LMN+APAP	M
control	-	**<0.001**	0.12	**0.04**	0.99	**<0.001**	**0.03**	**<0.001**
APAP		-	**<0.001**	**<0.001**	**<0.001**	0.91	**<0.001**	0.79
LMN			-	**<0.001**	0.08	**<0.001**	0.97	**<0.001**
LN				-	0.05	**<0.001**	**<0.001**	**<0.001**
LN+APAP					-	**<0.001**	0.07	**<0.001**
M+APAP						-	**<0.001**	0.99
LMN+APAP							-	**<0.001**
M								-
IL-6, pg/mL								
	control	APAP	LMN	LN	LN+APAP	M+APAP	LMN+APAP	M
control	-	**<0.001**	**<0.001**	**0.002**	**0.002**	**<0.001**	**<0.001**	**<0.001**
APAP		**-**	**<0.001**	**<0.001**	**<0.001**	**<0.001**	**<0.001**	**<0.001**
LMN			-	**<0.001**	**<0.001**	**0.011**	0.91	**0.015**
LN				-	0.96	**<0.001**	**<0.001**	**<0.001**
LN+APAP					-	**<0.001**	**<0.001**	**<0.001**
M+APAP						-	**0.03**	0.91
LMN+APAP							-	**0.04**
M								-
TNF-α, pg/mL								
	control	APAP	LMN	LN	LN+APAP	M+APAP	LMN+APAP	M
control	-	**<0.001**	0.99	**<0.001**	**<0.001**	**<0.001**	**<0.001**	**<0.001**
APAP		-	**<0.001**	**<0.001**	**<0.001**	**<0.001**	**<0.001**	**<0.001**
LMN			-	**<0.001**	**<0.001**	**<0.001**	**<0.001**	**<0.001**
LN				-	0.29	**<0.001**	0.88	**<0.001**
LN+APAP					-	**<0.001**	0.65	**<0.001**
M+APAP						-	**<0.001**	0.052
LMN+APAP							-	**<0.001**
M								-
IL-1β, ng/mL								
	control	APAP	LMN	LN	LN+APAP	M+APAP	LMN+APAP	M
control	-	**<0.001**	0.23	**<0.001**	**<0.001**	**<0.001**	0.21	**<0.001**
APAP		-	**<0.001**	**<0.001**	**<0.001**	0.36	**<0.001**	0.47
LMN			-	**<0.001**	**<0.001**	**<0.001**	0.99	**<0.001**
LN				-	0.62	**<0.001**	0.51	**<0.001**
LN+APAP					-	**<0.001**	0.44	**<0.001**
M+APAP						-	**<0.001**	0.53
LMN+APAP							-	**<0.001**
M								-

Values in bold are different from 0 with a significance level *p* < 0.05. APAP administration induced a robust inflammatory response, significantly increasing IL-6, TNF-α, and IL-1β levels and elevating the anti-inflammatory cytokine IL-10. LMN alone had minimal effects on basal cytokine levels. LMN+APAP consistently normalized cytokine responses, significantly reducing IL-6, TNF-α, and IL-1β, while modulating IL-10 toward control levels. LN, M, LN+APAP, and M+APAP produced only partial or inconsistent effects.

**Table 3:** Correlation matrix (Pearson’s *r*) showing relationships between oxidative stress markers, antioxidant enzymes, inflammatory cytokines, and liver enzymes.

Parameter	MDA	SOD	CAT	GPx	IL-10	IL-6	TNF-α	IL-1β	AST	ALT	ALP
MDA	1.000	−0.317	**0.844**	−0.754	0.320	0.779	−0.032	0.277	0.659	0.948	0.742
SOD		1.000	−0.404	0.757	−0.817	−0.787	−0.708	−0.638	−0.448	−0.393	−0.811
CAT			1.000	−0.581	0.463	0.804	0.135	0.521	**0.855**	**0.870**	0.808
GPx				1.000	−0.636	−0.904	−0.294	−0.317	−0.535	−0.695	−0.892
IL-10					1.000	0.665	**0.857**	0.741	0.445	0.465	0.735
IL-6						1.000	0.485	0.489	0.712	**0.848**	0.753
TNF-α							1.000	0.816	0.230	0.249	0.576
IL-1β								1.000	0.577	0.586	0.618
AST									1.000	**0.848**	**0.894**
ALT										1.000	**0.876**
ALP											1.000

Values in bold identify moderate to strong positive correlation (r > 0.840).

**Table 4:** Semi-quantitative scoring of liver histopathology (0–4).

Feature	0	1 (Mild)	2 (Moderate)	3 (Marked)	4 (Severe)
Hepatocellular Necrosis	None	Scattered individual cells	Centrilobular necrosis < 25% lobule	Centrilobular necrosis 25–50% lobule	Necrosis > 50% of lobule or panlobular
Inflammatory Infiltration	None	Minimal, focal	Moderate, multifocal	Marked, bridging areas	Severe, diffuse with aggregates
Vacuolization/Fatty Degeneration	None	Microvesicles < 10% hepatocytes	10–25% hepatocytes	25–50% hepatocytes	50% hepatocytes, macrovesicles

**Table 5:** Comparative assessment of representative flavonoid-loaded nanoparticle delivery systems used in the literature, based on their physicochemical features, biological performance, and limitations to contextualize the distinct properties of LMN applied in the present study.

Criteria	LMN (Present Study)	Flavonoid-Loaded Chitosan-Alginate-Polysaccharide NPs [74]	Flavonoid-Loaded PLGA/Polyester NPs [70]	Lipid-Based Systems (Nanoemulsions, Self-Nanoemulsifying Drug Delivery Systems, and Liposomes) [75,76,77,78]	Flavonoid-Loaded Mesoporous Silica NPs [79,80]
origin and composition of the carrier	alkali lignin—renewable aromatic biopolymer rich in phenolic groups	natural polysaccharides; cationic (chitosan) or anionic (alginate), mucoadhesive	synthetic biodegradable polyesters PLGA, PLA poly-lactic acid (PLA), poly-ε-caprolactone (PCL)	lipids, surfactants, oils; generally recognized as safe (GRAS) excipients	inorganic silica with mesoporous structure; tunable pore size
carrier intrinsic antioxidant activity	high radical scavenging activity of lignin towards ROS; addition effect with morin-unique advantage	moderate chitosan mild radical scavenging potential; minimal antioxidant activity of alginate	no inherent redox activity	none—inert carriers	none—inert carrier with possibility for functionalization
compatibility with polyphenols	excellent due to π–π and hydrogen bonding interactions; high loading	good—electrostatic interactions; moderate loading	good but sometimes limited by drug polarity	excellent for lipophilic flavonoids; solubilization-driven	excellent—pore adsorption enables high loading
nanoparticle stability	stable in aqueous media	stable at neutral pH; chitosan dissolves at low pH unless crosslinked	very stable; slow hydrolysis	less stable; possible phase separation	very stable inorganic shells
release behavior	sustained release	slow release if crosslinked; initial burst effect possible	controlled release; tunable with polymer molecular weight	fast release; tuned mostly by oil/water partition	controlled diffusion from pores; tunable via pore size
mechanisms relevant to APAP protection	Dual: (i) morin antioxidant + anti-inflammatory; (ii) lignin’s ROS scavenging and phenoxy stabilization	flavonoid-mediated antioxidant effects; moderate contribution of the carrier	flavonoid-mediated; controlled release improves plasma exposure	enhanced absorption leading to increased systemic antioxidant levels	concentrated delivery of flavonoids to the liver, spleen, and lymph nodes
evidence in APAP toxicity models	limited—LMN not yet broadly reported	strong evidence for quercetin, naringenin, galangin NPs reducing APAP injury	limited evidence	limited APAP data; more evidence on pharmacokinetic enhancement	sparse; more evidence in cancer/oxidative injury
biodistribution pattern of the flavonoid	liver > kidney > spleen	moderate liver uptake; fast clearance	high early liver accumulation, then clearance over 48–72 h	systemic distribution mainly; organ levels depend on lipid solubility	strong liver/spleen accumulation due to inorganic NP capture
scalability and cost	excellent—lignin is low-cost and abundant precursor	good	moderate but industrially scalable	excellent—food/pharma lipids	variable
environmental sustainability	very high—based on renewable lignocellulosic waste	high—biopolymers	moderate	high	low
advantages	addition effect antioxidant carrier + active drug; sustainable; high liver targeting	biocompatible; good for oral delivery; mucoadhesive	predictable release; FDA approved; strong stability	improved solubility and absorption; simple	high loading; tunable porous structure
limitations	particle heterogeneity	lower loading; pH-sensitive	expensive; hydrophobic matrix may slow release	instability; limited organ targeting	long-term inorganic accumulation concerns

**Table 6:** Experimental groups and treatment regimens.

Group	n	Treatment	Dose	Duration
1—Control	6	Saline (i.p.)	—	3 days
2—Acetaminophen (APAP)	6	APAP (i.p.)	200 mg/kg	3 days
3—Morin (M)	6	M (i.p.)	50 mg/kg	5 days
4—Lignin nanoparticles (LN)	6	LN (i.p.)	50 mg/kg	5 days
5—Lignin/Morin nanoparticles (LMN)	6	LMN (i.p.)	50 mg/kg	5 days
6—Morin followed by APAP (M+APAP)	6	M (i.p.) → APAP (i.p.)	50 mg/kg → 200 mg/kg	5 days → 3 days
7—LN followed by APAP (LN+APAP)	6	LN (i.p.) → APAP (i.p.)	50 mg/kg → 200 mg/kg	5 days → 3 days
8—LMN followed by APAP (LMN+APAP)	6	LMN (i.p.) → APAP (i.p.)	50 mg/kg → 200 mg/kg	5 days → 3 days

## Data Availability

The original contributions presented in this study are included in the article/Supplementary Material. Further inquiries can be directed to the corresponding author.

## Supplementary Materials

The following supporting information can be downloaded at https://www.mdpi.com/article/10.3390/ph19010071/s1, Table S1: Tukey post hoc *p*-value matrix for the MDA, CAT, SOD, and GPx activity values.

## References

[1] Liao J., Lu Q., Li Z., Li J., Zhao Q., Li J. (2023). Acetaminophen-induced liver injury: Molecular mechanism and treatments from natural products. Front. Pharmacol..

[2] Hionides-Gutierrez A., Goikoetxea-Usandizaga N., Sanz-García C., Martínez-Chantar M.L., Cubero F.J. (2025). Novel Emerging Mechanisms in Acetaminophen (APAP) Hepatotoxicity. Liver Int..

[3] Yao Q., Tang Y., Dai S., Huang L., Jiang Z., Zheng S., Sun M., Xu Y., Lu R., Sun T. (2023). A Biomimetic Nanoparticle Exerting Protection against Acute Liver Failure by Suppressing CYP2E1 Activity and Scavenging Excessive ROS. Adv. Healthc. Mater..

[4] Pancheva R., Dolinsek J., Panayotova M., Yankov I., Kofinova D., Nikolova S., Baycheva M., Georgieva M. (2025). Bridging the Gap: Awareness, Knowledge, and Challenges of Living with Celiac Disease in Bulgaria. Nutrients.

[5] Panayotova M., Penkova M. (2024). Measurement of oxidative stress-related markers in gastro-intestinal damages in Bulgarian pediatric patients. Bulg. Chem. Commun..

[6] Shen X.L., Guo Y.N., Lu M.H., Ding K.N., Liang S.S., Mou R.W., Yuan S., He Y.M., Tang L.P. (2023). Acetaminophen-Induced Hepatotoxicity Predominantly via Inhibiting Nrf2 Antioxidative Pathway and Activating TLR4-NF-κB-MAPK Inflammatory Response in Mice. Ecotoxicol. Environ. Saf..

[7] Nogué-Xarau S., Martínez-Sánchez L., García-Peláez M., Fernández de Gamarra-Martínez E., Pi-Sala N., Gispert-Ametller À., Salgado-García E., Aguilar-Salmerón R. (2025). N-acetylcysteine: 50 years since the discovery of an antidote that has changed the prognosis of acetaminophen poisoning. Farm. Hosp..

[8] Patel J., Roy H., Chintamaneni P.K., Patel R., Bohara R. (2025). Advanced Strategies in Enhancing the Hepatoprotective Efficacy of Natural Products: Integrating Nanotechnology, Genomics, and Mechanistic Insights. ACS Biomater. Sci. Eng..

[9] Sahibzada M.U.K., Sadiq A., Zahoor M., Naz S., Shahia M., Qureshi N.A. (2020). Enhancement of bioavailability and hepatoprotection by silibinin through conversion to nanoparticles prepared by liquid antisolvent method. Arab. J. Chem..

[10] Vanholme R., De Meester B., Ralph J., Boerjan W. (2019). Lignin biosynthesis and its integration into metabolism. Curr. Opin. Biotechnol..

[11] Pereira A.E.S., de Oliveira J.L., Savassa S.M., Rogério C.B., de Medeiros G.A., Fraceto L.F. (2022). Lignin Nanoparticles: New Insights for a Sustainable Agriculture. J. Clean. Prod..

[12] Sadeghifar H., Ragauskas A.J. (2025). Lignin as a Natural Antioxidant: Chemistry and Applications. Macromol.

[13] Huo C.M., Zuo Y.C., Chen Y., Chen L., Zhu J.Y., Xue W. (2024). Natural Lignin Nanoparticles Target Tumor by Saturating the Phagocytic Capacity of Kupffer Cells in the Liver. Int. J. Biol. Macromol..

[14] Mehta J., Kumar P., Pawar S.V. (2025). Exploration of Capsaicin-Encapsulated Lignin Nanoparticles for Alleviating Non-Alcoholic Fatty Liver Disease: In-Vitro Study. Int. J. Biol. Macromol..

[15] Porto D.S., Estevão B.M., Lins P.M.P., Rissi N.C., Zucolotto V., da Silva M.F.G.F. (2021). Orange Trunk Waste-Based Lignin Nanoparticles Encapsulating Curcumin as a Photodynamic Therapy Agent against Liver Cancer. ACS Appl. Polym. Mater..

[16] Pylypchuk I.V., Suo H., Chucheepchuenkamol C., Jedicke N., Lindén P.A., Lindström M.E., Manns M.P., Sevastyanova O., Yevsa T. (2022). High-Molecular-Weight Fractions of Spruce and Eucalyptus Lignin as a Perspective Nanoparticle-Based Platform for Therapy Delivery in Liver Cancer. Front. Bioeng. Biotechnol..

[17] Yu H., Wang B., Wang Y., Xu E., Wang R., Wu S., Wu W., Ji B., Feng X., Xu H. (2023). Lignin Nanoparticles with High Phenolic Content as Efficient Antioxidant and Sun-Blocker for Food and Cosmetics. ACS Sustain. Chem. Eng..

[18] Petkova-Parlapanska K., Nikolova G., Karamalakova Y., Nicheva D., Rusenova N., Beev G., Georgieva E., Yanev Ž., Georgieva D., Hristova S., Petkov P., Achour M.E., Popov C. (2025). Sustainable Lignin Nanoparticles: A Versatile Proxy Agent with Advanced Environmental and Biomedical Significance. Nanotechnological Advances in Environmental, Cyber and CBRN Security.

[19] Yanev Z., Georgieva D., Hristova S., Tzanova M., Nicheva D., Andonova-Lilova B., Zagorcheva T., Vladova D., Grozeva N., Yaneva Z. (2025). Innovative In Situ Interfacial Co-Assembled Lignin/Chitosan Nanoparticles—Green Synthesis, Physicochemical Characterization, In Vitro Release, and Intermolecular Interactions. Int. J. Mol. Sci..

[20] Ivanova D., Toneva M., Simeonov E., Nikolova B., Semkova S., Antov G., Yaneva Z. (2023). Newly Synthesized Lignin Microparticles as Bioinspired Oral Drug-Delivery Vehicles: Flavonoid-Carrier Potential and In Vitro Radical-Scavenging Activity. Pharmaceutics.

[21] Ding Y., Li Q., Xu Y., Chen Y., Deng Y., Zhi F., Qian K. (2016). Attenuating oxidative stress by paeonol protected against acetaminophen-induced hepatotoxicity in mice. PLoS ONE.

[22] Al-Doaiss A.A. (2020). Hepatotoxicity induced by the therapeutic dose of acetaminophen and the ameliorative effect of oral co-administration of selenium/*Tribulus terrestris* extract in rats. Int. J. Morphol..

[23] Kane A.E., Mitchell S.J., Mach J., Huizer-Pajkos A., McKenzie C., Jones B., Cogger V., Le Couteur D.G., de Cabo R., Hilmer S.N. (2016). Acetaminophen hepatotoxicity in mice: Effect of age, frailty and exposure type. Exp. Gerontol..

[24] Mao J., Tan L., Tian C., Wang W., Zhang H., Zhu Z., Li Y. (2024). Research progress on rodent models and mechanisms of liver injury. Life Sci..

[25] Papáčková Z., Heczkova M., Dankova H., Sticova E., Lodererova A., Bartonova L., Poruba M., Cahova M. (2018). Silymarin prevents acetaminophen-induced hepatotoxicity in mice. PLoS ONE.

[26] Chowdhury A., Lu J., Zhang R., Nabila J., Gao H., Wan Z., Temitope I.A., Yin X., Sun Y. (2019). Mangiferin ameliorates acetaminophen-induced hepatotoxicity in mice by improving metabolism and reducing oxidative stress and inflammation. Biomed. Pharmacother..

[27] Zhang X., He Y., Chen Y., Luo L., Zhang Y., Ma G. (2023). Engineered Nanoparticles for Liver-Targeted Drug Delivery: Modulation of Kupffer Cell Uptake and Biodistribution. J. Control Release.

[28] Penalva R., González-Navarro C.J., Gamazo C., Esparza I., Irache J.M. (2017). Zein nanoparticles for oral delivery of quercetin: Pharmacokinetic studies and preventive anti-inflammatory effects in a mouse model of endotoxemia. Nanomedicine.

[29] Ding C., Zhao Y., Chen X., Zheng Y., Liu W., Liu X. (2021). Taxifolin, a Novel Food, Attenuates Acute Alcohol-Induced Liver Injury in Mice through Regulating the NF-κB-Mediated Inflammation and PI3K/Akt Signalling Pathways. Pharm. Biol..

[30] Meng B., Zhang Y., Wang Z., Ding Q., Song J., Wang D. (2019). Hepatoprotective Effects of *Morchella esculenta* against Alcohol-Induced Acute Liver Injury in the C57BL/6 Mouse Related to Nrf-2 and NF-κB Signaling. Oxidative Med. Cell Longev..

[31] Bhakuni G.S., Bedi O., Bariwal J., Kumar P. (2017). Hepatoprotective Activity of Morin and Its Semi-Synthetic Derivatives against Alcohol-Induced Hepatotoxicity in Rats. Indian J. Physiol. Pharmacol..

[32] Yaneva Z., Ivanova D., Toneva M. (2024). Green Synthesis, Characterization, Encapsulation, and Measurement of the Release Potential of Novel Alkali Lignin Micro-/Submicron Particles. J. Vis. Exp..

[33] Ren Y., Xu Z., Qiao Z., Wang X., Yang C. (2024). Flaxseed Lignan Alleviates the Paracetamol-Induced Hepatotoxicity Associated with Regulation of Gut Microbiota and Serum Metabolome. Nutrients.

[34] Kaur H., Goyal D. (2024). Lignin Extraction from Lignocellulosic Biomass and Its Valorization to Therapeutic Phenolic Compounds. J. Environ. Manag..

[35] Orlov A., Semenov S., Rukhovich G., Sarycheva A., Kovaleva O., Semenov A., Ermakova E., Gubareva E., Bugrova A.E., Kononikhin A. (2022). Hepatoprotective Activity of Lignin-Derived Polyphenols Dereplicated Using High-Resolution Mass Spectrometry, In Vivo Experiments, and Deep Learning. Int. J. Mol. Sci..

[36] Pandi A., Sen N., Manickam Kalappan V., Lal V. (2025). Morin: A Promising Hepatoprotective Agent against Drug and Chemical-Induced Liver Injury. Naunyn-Schmiedeberg’s Arch. Pharmacol..

[37] Gendy A., Elnagar M.R., Soubh A., Al-Mokaddem A., El-Haddad A., El-Sayed M.K. (2021). Morin Alleviates Hepatic Ischemia/Reperfusion-Induced Mischief: In Vivo and In Silico Contribution of Nrf2, TLR4, and NLRP3. Biomed. Pharmacother..

[38] Sheoran R., Bhardwaj S., Phogat A., Hasanpuri A., Malik V. (2025). Hepatoprotective Effect of Morin Against Lead-Induced Oxidative Stress and Structural Alterations in Male Wistar Rats: An In Vivo and In Silico Approach. J. Appl. Toxicol..

[39] Shali K.S., Soumya N.P., Mondal S., Mini S. (2022). Hepatoprotective Effect of Morin via Regulating the Oxidative Stress and Carbohydrate Metabolism in Streptozotocin-Induced Diabetic Rats. Bioactive Compounds Health Dis..

[40] Folorunso I.M., Lawal A.O., Elekofehinti O.O., Oyetayo O.S., Omotuyi I.O., Oduola T., Adelakun O.E., Olorunfemi N.O., Ojo E.O., Ajiboye B. (2023). Hepatoprotective Effect of Morin Hydrate in Type 2 Diabetic Wistar Rats Exposed to Diesel Exhaust Particles. Appl. Biochem. Biotechnol..

[41] Mondal S., Das S., Mahapatra P.K., Saha K.D. (2022). Morin-Vitamin E-β-Cyclodextrin Inclusion Complex-Loaded Chitosan Nanoparticles (M-Vit.E-CD-CSNPs) Ameliorate Arsenic-Induced Hepatotoxicity in a Murine Model. Molecules.

[42] Gu J., Wu J., Xu S., Zhang L., Fan D., Shi L., Wang J., Ji G. (2020). Bisphenol F Exposure Impairs Neurodevelopment in Zebrafish Larvae (*Danio rerio*). Ecotoxicol. Environ. Saf..

[43] Hossen M.S., Akter A., Azmal M., Rayhan M., Islam K.S., Islam M.M., Ahmed S., Abdullah-Al-Shoeb M. (2024). Unveiling the Molecular Basis of Paracetamol-Induced Hepatotoxicity: Interaction of N-Acetyl-p-Benzoquinone Imine with Mitochondrial Succinate Dehydrogenase. Biochem. Biophys. Rep..

[44] Pei J., Pan X., Wei G., Hua Y. (2023). Research Progress of Glutathione Peroxidase Family (GPX) in Redoxidation. Front. Pharmacol..

[45] Deng J., Gu J., Zhao X., Yan B., Wang L., Ji G., Huang C. (2023). Improving the Protective Ability of Lignin against Vascular and Neurological Development in BPAF-Induced Zebrafish by High-Pressure Homogenization Technology. Int. J. Biol. Macromol..

[46] Liang R., Zhao J., Li B., Cai P., Loh X.J., Xu C., Chen P., Kai D., Zheng L. (2020). Implantable and Degradable Antioxidant Poly (ε-Caprolactone)-Lignin Nanofiber Membrane for Effective Osteoarthritis Treatment. Biomaterials.

[47] Fedoros E.I., Baldueva I.A., Perminova I.V., Badun G.A., Chernysheva M.G., Grozdova I.D., Melik-Nubarov N.S., Danilova A.B., Nekhaeva T.L., Kuznetsova A.I. (2020). Exploring Bioactivity Potential of Polyphenolic Water-Soluble Lignin Derivative. Environ. Res..

[48] Jali A.M., Alam M.F., Hanbashi A., Mawkili W., Abdlasaed B.M., Alshahrani S., Qahl A.M., Alrashah A.S.S., Shahi H.A. (2023). Sesamin’s Therapeutic Actions on Cyclophosphamide-Induced Hepatotoxicity, Molecular Mechanisms, and Histopathological Characteristics. Biomedicines.

[49] Kamal G.F., Nasr M.Y., Hussein M.A., Abdel-Aziz A., Fayed A.M. (2022). Morin: A Promising Nutraceutical Therapy for Modulation of the NF-κB/NOX-2/IL-6/HO-1 Signaling Pathways in Paracetamol-Induced Liver Toxicity. Biomed. Res. Ther..

[50] Kumar V., Kumar R., Gurusubramanian G., Rathore S.S., Roy V.K. (2024). Morin Hydrate Ameliorates Di-2-Ethylhexyl Phthalate (DEHP)-Induced Hepatotoxicity in a Mouse Model via TNF-α and NF-κB Signaling. 3 Biotech.

[51] Gheena S., Ezhilarasan D., Shree Harini K., Rajeshkumar S. (2022). Syringic Acid and Silymarin Concurrent Administration Inhibits Sodium Valproate-Induced Liver Injury in Rats. Environ. Toxicol..

[52] Mondal S., Das S., Mahapatra P.K., Saha K. (2022). Morin-Encapsulated Chitosan Nanoparticles (MCNPs) Ameliorate Arsenic-Induced Liver Damage through Improvement of the Antioxidant System and Prevention of Apoptosis and Inflammation in Mice. Nanoscale Adv..

[53] Campbell F., Bos F.L., Sieber S., Arias-Alpizar G., Koch B.E., Huwyler J., Kros A., Bussmann J. (2018). Directing Nanoparticle Biodistribution through Evasion and Exploitation of Stab2-Dependent Nanoparticle Uptake. ACS Nano.

[54] Zapotoczny B., Szafrańska K., Lekka M., Ahluwalia B.S., McCourt P. (2022). Tuning of Liver Sieve: The Interplay between Actin and Myosin Regulatory Light Chain Regulates Fenestration Size and Number in Murine Liver Sinusoidal Endothelial Cells. Int. J. Mol. Sci..

[55] Yaneva Z.L., Georgieva N.V., Bekirska L.L., Lavrova S. (2018). Drug Mass Transfer Mechanism, Thermodynamics, and In Vitro Release Kinetics of Antioxidant-Encapsulated Zeolite Microparticles as a Drug Carrier System. Chem. Biochem. Eng. Q..

[56] Yaneva Z., Ivanova D., Toneva M., Tzanova M., Marutsova V., Grozeva N. (2023). Menadione Contribution to the In Vitro Radical Scavenging Potential of Phytochemicals Naringenin and Lignin. Int. J. Mol. Sci..

[57] Yaneva Z., Beev G., Rusenova N., Ivanova D., Tzanova M., Stoeva D., Toneva M. (2022). Antimicrobial Potential of Conjugated Lignin/Morin/Chitosan Combinations as a Function of System Complexity. Antibiotics.

[58] Toneva M.R., Ivanova D.G., Tzanova M.T., Yaneva Z.L. (2024). In Vitro and In Vivo Evaluation of the Multifaceted Physiological Roles and Biochemical Pathways of Lignin- and Morin-Based Formulations. Bulgar. Chem. Commun..

[59] Yaneva Z., Ivanova D., Nikolova G., Karamalakova Y., Rusenova N., Georgieva E., Petkova-Parlapanska K. (2024). Nanoparticle Compositions. GB Patent.

[60] Pacorig V., Galeotti M., Beraldo P. (2022). Multiparametric semi-quantitative scoring system for the histological evaluation of marine fish larval and juvenile quality. Aquac. Rep..

[61] Jaeschke H., Ramachandran A. (2024). Paradigm for Understanding Mechanisms of Drug-Induced Liver Injury. Annu. Rev. Pathol..

[62] Brzhozovskiy A.G., Semenov S.D., Zherebker A.Y., Bugrova A.E., Yurova M.N., Zhernov Y.V., Kovaleva O.A., Semenov A.L., Abroskin D.P., Kruglov S.S. (2025). Hepatoprotective Activity of Nature-Derived Polyphenols Studied by Mass Spectrometry Based Multi-OMICS Approach. Int. J. Mol. Sci..

[63] Ohashi N., Kohno T. (2020). Analgesic Effect of Acetaminophen: A Review of Known and Novel Mechanisms of Action. Front. Pharmacol..

[64] Sivagurunathan N., Calivarathan L. (2025). Inflammasome Activation as a Key Driver of Acetaminophen-Induced Hepatotoxicity: Mechanisms and Emerging Therapeutics. Gene Expr..

[65] Luo G., Huang L., Zhang Z. (2023). The Molecular Mechanisms of Acetaminophen-Induced Hepatotoxicity and Its Potential Therapeutic Targets. Exp. Biol. Med..

[66] Ramachandran A., Jaeschke H. (2024). Clinically Relevant Therapeutic Approaches against Acetaminophen Hepatotoxicity and Acute Liver Failure. Biochim. Biophys. Acta Mol. Basis Dis..

[67] Chang L., Xu D., Zhu J., Ge G., Kong X., Zhou Y. (2020). Herbal Therapy for the Treatment of Acetaminophen-Associated Liver Injury: Recent Advances and Future Perspectives. Front. Pharmacol..

[68] Zhao E., Liang R., Li P., Liu X., Xu Y., Chen Z., Xu C., Zhang Y., Liu J., Zhuang Y. (2024). Mesenchymal Stromal Cells Alleviate APAP-Induced Liver Injury via Extracellular Vesicle-Mediated Regulation of the miR-186-5p/CXCL1 Axis. Stem Cell Res. Ther..

[69] Ivanova D., Nikolova G., Karamalakova Y., Semkova S., Marutsova V., Yaneva Z. (2023). Water-Soluble Alkali Lignin as a Natural Radical Scavenger and Anticancer Alternative. Int. J. Mol. Sci..

[70] Rizvi F., Mathur A., Kakkar P. (2015). Morin mitigates acetaminophen-induced liver injury by potentiating Nrf2 regulated survival mechanism through molecular intervention in PHLPP2-Akt-Gsk3β axis. Apoptosis.

[71] Alonso M., Barcia E., González J.F., Montejo C., García-García L., Villa-Hermosilla M.C., Negro S., Fraguas-Sánchez A.I., Fernández-Carballido A. (2022). Functionalization of morin-loaded PLGA nanoparticles with phenylalanine dipeptide targeting the brain. Pharmaceutics.

[72] Rout G.K., Singh K.R.B., Mahari S., Jena A.B., Panigrahi B., Pradhan K.C., Pal S., Kisan B., Dandapat J., Singh J. (2023). Bioactive potential of morin-loaded mesoporous silica nanoparticles: A novel and efficient antioxidant, antidiabetic and biocompatible agent in in-silico, in-vitro and in-vivo models. OpenNano.

[73] Mao J., Liu X., Zhang L., Chen Y., Zhou S., Liu Y., Ye J., Xu X., Zhang Q. (2024). Self-nanoemulsifying drug delivery system of morin: A new approach for combating acute alcohol intoxication. Int. J. Nanomed..

[74] Tzankova V., Aluani D., Kondeva-Burdina M., Yordanov Y., Odzhakov F., Apostolov A., Yoncheva K. (2017). Hepatoprotective and antioxidant activity of quercetin-loaded chitosan/alginate particles in vitro and in vivo in a model of paracetamol-induced toxicity. Biomed. Pharmacother..

[75] Son H.-Y., Lee M.-S., Chang E., Kim S.-Y., Kang B., Ko H., Kim Y.-H. (2019). Formulation and characterization of quercetin-loaded oil-in-water nanoemulsion and evaluation of hypocholesterolemic activity in rats. Nutrients.

[76] Das S.S., Sarkar A., Chabattula S.C., Verma P.R.P., Nazir A., Gupta P.K., Ruokolainen J., Kesari K.K., Singh S.K. (2022). Food-grade quercetin-loaded nanoemulsion ameliorates effects associated with Parkinson’s disease and cancer: Studies employing a transgenic C. elegans model and human cancer cell lines. Antioxidants.

[77] Li H., Chen P., Wang M., Wang W., Li F., Han X., Ren J., Duan X. (2022). Liposome quercetin enhances the ablation effects of microwave ablation in treating the rabbit VX2 liver tumor model. Int. J. Hyperth..

[78] Ranjbar S., Emamjomeh A., Sharifi F., Zarepour A., Aghaabbasi K., Dehshahri A., Sepahvand A.M., Zarrabi A., Beyzaei H., Zahedi M.M. (2023). Lipid-based delivery systems for flavonoids and flavonolignans: Liposomes, nanoemulsions and solid lipid nanoparticles. Pharmaceutics.

[79] Arriagada F., Günther G., Morales J. (2020). Nanoantioxidant-based silica particles as flavonoid carriers for drug-delivery applications. Pharmaceutics.

[80] Elmowafy M., Alruwaili N.K., Ahmad N., Kassem A.M., Ibrahim M.F. (2022). Quercetin-loaded mesoporous silica nanoparticle-based lyophilized tablets for enhanced physicochemical features and dissolution rate: Formulation, optimization and in vitro evaluation. AAPS PharmSciTech.

[81] Rohrer P.R., Rudraiah S., Goedken M.J., Manautou J.E. (2014). Is nuclear factor erythroid 2-related factor 2 responsible for sex differences in susceptibility to acetaminophen-induced hepatotoxicity in mice?. Drug Metab. Dispos..

[82] Regulation № 20/01.11.2012 of the Minimum Requirements for the Protection and Welfare of Experimental Animals and the Sites Used for Their Breeding, Use, or Delivery. https://lex.bg/laws/ldoc/2135820878.

[83] Sun Y., Oberley L.W., Li Y. (1988). A Simple Method for Clinical Assay of Superoxide Dismutase. Clin. Chem..

[84] Aebi H. (1984). Catalase in vitro. Meth. Enzymol..

[85] Placer Z.A., Cushman L.L., Johnson B.C. (1966). Estimation of Product of Lipid Peroxidation (Malonyl Dialdehyde) in Biochemical Systems. Anal. Biochem..

[86] Karamalakova Y.D., Nikolova G.D., Zheleva A.M., Gadjeva V.G. (2020). An Investigation on the Antioxidative Properties of Glycine Amide Derivative of 2,2,6,6-Tetramethyl-4-Aminopiperidine-1-Oxyl Affecting Pancreas Protection. J. Chem. Technol. Metall..

[87] Sharma D., Singh M., Kumar P., Vikram V., Mishra N. (2017). Development and characterization of morin hydrate–loaded microemulsion for the management of Alzheimer’s disease. Artif. Cells Nanomed. Biotechnol..

