# Supplementary material for: Ameliorated Hepatoprotective Aptitude of Novel Lignin Nanoparticles on APAP-Induced Hepatotoxicity in a Murine Model
Source: Pharmaceuticals (Basel). 2025 Dec 29;19(1):71. doi: 10.3390/ph19010071 (PMC12844817; doi:10.3390/ph19010071)
Supplement: Supplementary file 1 [file pharmaceuticals-19-00071-s001.zip › Table S1.pdf]

Table S1. Tukey post-hoc p-value matrix for the MDA, CAT, SOD and GPx activity values: APAP group differs significantly from all others ($p < 0.001$), confirming strong oxidative stress regarding the four parameters. MDA values: LMN+APAP, LN+APAP, and M+APAP show highly significant improvements vs APAP ($p < 0.001$). LN and LMN alone are not significantly different from control ($p > 0.05$), indicating biocompatibility. M is significantly different from most groups ($p < 0.001$), reflecting potent antioxidant activity. SOD activity: LN, LN+APAP, and LMN treatments significantly improve SOD activity vs APAP. LMN+APAP and M+APAP show strong recovery ($p < 0.01$ vs APAP), though still lower than control. M improves SOD moderately but remains below control levels. LN and LN+APAP are statistically close ($p > 0.05$), showing similar antioxidant trends CAT activity: All antioxidant co-treatments (LMN+APAP, LN+APAP, M+APAP) reduce CAT levels toward normal (non-significant difference vs control). Pure LMN, LN, and M groups do not differ significantly from control, confirming biocompatibility and homeostatic effects. LN+APAP shows mild significance ($p \approx 0.05$), suggesting slightly stronger modulation of CAT among combination treatments.**GPx values:** LN, LN+APAP, and M groups show GPx levels close to control ($p > 0.05$), demonstrating strong recovery. LMN+APAP and M+APAP significantly improve GPx vs APAP ($p < 0.01$), but remain somewhat below full normalization. LMN alone increases GPx relative to APAP but still differs from control ($p < 0.001$), reflecting moderate protective effect.

Group	control	APAP	LMN	LN	LN+APAP	M+APAP	LMN+APAP	M
				**MDA**				
control	-	**<0.001**	**<0.001**	0.642	**<0.001**	**<0.001**	**<0.001**	**<0.001**
APAP		-	**<0.001**	**<0.001**	**<0.001**	**<0.001**	**<0.001**	**<0.001**
LMN			-	**<0.001**	**<0.001**	0.218	**<0.001**	**<0.001**
LN				-	**<0.001**	**<0.001**	**<0.001**	**<0.001**
LN+APAP					-	**<0.001**	0.776	**<0.001**
M+APAP						-	**<0.001**	**<0.001**
LMN+APAP							-	**<0.001**
M								-
				**SOD**				
control	-	**<0.001**	**<0.001**	**0.017**	**0.028**	**<0.001**	**<0.001**	**<0.001**
APAP		-	**0.002**	**<0.001**	**<0.001**	**0.036**	0.054	0.546
LMN			-	0.094	0.142	**0.003**	**0.002**	**0.004**
LN				-	0.523	**<0.001**	**<0.001**	**<0.001**
LN+APAP					-	**<0.001**	**<0.001**	**<0.001**
M+APAP						-	0.423	0.118
LMN+APAP							-	0.091
M								-
				**CAT**				
control	-	**<0.001**	0.421	0.762	0.049	0.363	0.408	0.687
APAP		-	**<0.001**	**<0.001**	**<0.001**	**<0.001**	**<0.001**	**<0.001**
LMN			-	0.408	0.094	0.829	0.923	0.514
LN				-	0.071	0.626	0.592	0.894
LN+APAP					-	0.213	0.244	0.085
M+APAP						-	0.884	0.602

LMN+APAP							-	0.575
M								-
				**GPx**				
control	-	**<0.001**	**<0.001**	0.428	0.063	**<0.001**	**<0.001**	**0.012**
APAP		-	**<0.001**	**<0.001**	**<0.001**	**0.001**	**0.002**	**<0.001**
LMN			-	**<0.001**	**<0.001**	0.112	0.139	**0.007**
LN				-	0.093	**<0.001**	**<0.001**	**0.027**
LN+APAP					-	**<0.001**	**<0.001**	0.054
M+APAP						-	0.734	**0.016**
LMN+APAP							-	**0.021**
M								-

p < 0.05 – significant; p < 0.01 - very significant; p < 0.001 **-** highly significant
